# Fully integrated quantum frequency processor on a silicon chip

**DOI:** 10.1038/s41467-026-77287-5

**Published:** 2026-09-15

**Authors:** Sara Congia, Leopold Virot, Elena Rovetta, Antonio Fincato, Frederic Boeuf, Matteo Galli, Daniele Bajoni, Massimo Borghi

**Affiliations:** 1 Dipartimento di Fisica “Alessandro Volta”, Università di Pavia, via Bassi 6, Pavia, Italy; 2 Université Grenoble Alpes, CEA-Leti, Grenoble, France; 3 STMicroelectronics, Crolles, France; 4 Dipartimento di Ingegneria Industriale e dell’Informazione, Università di Pavia, via Ferrata 1, Pavia, Italy; 5 Present Address: Dipartimento di Ingegneria Industriale e dell’Informazione, Università di Pavia, via Ferrata 1, Pavia, Italy; 6 Present Address: STMicroelectronics, Crolles, France

**Keywords:** Frequency combs, Quantum information

## Abstract

Frequency-bin encoding has recently emerged as a powerful approach for photonic quantum information processing, offering high dimensionality, gate-parallelization, and compatibility with existing telecommunication infrastructure. However, its scalable deployment has so far been hindered by the lack of an integrated platform capable of unifying quantum state generation, coherent frequency mixing, and programmable spectral control. Here, we report a fully integrated quantum frequency processor, monolithically integrating on the same silicon photonic chip a microresonator-based biphoton quantum frequency comb source, a pump-rejection filter, high-speed phase modulators, and a four-channel, line-by-line pulse shaper. We demonstrate key functionalities, such as tunable frequency beamsplitters with success probabilities exceeding 94% and fidelities above 99.9%, as well as the ability to synthesize more general single-qubit gates. Finally, we generate and coherently manipulate high-dimensional frequency-bin entangled states entirely on chip, showcasing control over two-photon quantum walks and performing on-chip frequency-bin quantum state tomography of a Bell-state with a fidelity of 95.7(3)%. By integrating all key functional elements on the same 4 × 7 mm^2^ chip, with the possibility of scaling to a larger number of modes, our work marks an important step toward large-scale frequency-domain photonic processors for both classical and quantum applications.

## Abstract (web summary)

Frequency-bin encoding offers high-dimensional quantum information processing, but scalable deployment requires miniaturization. Here, the authors demonstrate quantum state generation and coherent manipulation on a CMOS-compatible photonic processor.

## Introduction

Integrated optical processors are chip-scale systems that generate, coherently manipulate, and transmit information using light^1–3^. To date, the most advanced photonic processors for classical data processing–in terms of integration density, reliability, performance, and component count–are capable of flexibly exploiting multiple optical degrees of freedom (DoF). Through large-scale programmable networks of beamsplitters and phase shifters, spectral filters, multimode waveguides, optical delay lines, and high-speed modulators, these systems can indiscriminately manipulate spatial, spectral, temporal, and polarization DoFs, enabling coherent transmission as well as analog optical and microwave signal processing^1,2,4–7^.

In sharp contrast, large-scale integrated quantum photonic processors predominantly rely on dual-rail or, more generally, multi-rail encodings^8–11^, a design choice largely driven by the need to manipulate fragile quantum states of light with minimal loss and high operational fidelity using mature linear-optical interferometric networks. While such encoding is well-suited for on-chip processing, it is intrinsically inefficient for quantum state distribution and for scaling to high-dimensional Hilbert spaces, as it requires multiple spatial modes. In this context, alternative photonic DoFs–such as time-bin^12,13^, transverse-mode^14,15^, and polarization^16,17^–are particularly attractive and are objects of active research, as they are naturally compatible with fiber-based transmission and enable high-dimensional encoding within a single spatial mode.

Recently, the frequency degree of freedom–traditionally used for multiplexing in both classical and quantum networks–has emerged as a basis for encoding quantum information itself^18^. In this framework, logical states are defined over discrete, narrowband, and non-overlapping spectral intervals, referred to as frequency bins (FBs)^19–21^. FB encoding possess compelling features, including single-spatial-mode operation, very high dimensionality^22^, gate parallelization^23,24^, and compatibility with existing telecommunication infrastructure^25^. Moreover, this encoding is naturally matched to on-chip quantum light sources based on biphoton frequency combs (BPFC) generated in optical microcavities^26^. Over the past few years, FB quantum information processing has witnessed significant progress, driven by both theoretical studies^18,23,27,28^ and experimental demonstrations, including quantum state engineering^29,30^, tomography^31^, Hong-Ou-Mandel interference^32^, metrology^33,34^, quantum walks^35,36^, quantum key distribution^37–39^, and continuous-variable cluster-state generation^21,40^.

An important step toward a complete framework for quantum information processing with FBs was established by Lukens and coworkers^18^, who introduced the Quantum Frequency Processor (QFP) as a fundamental processing unit combining coherent frequency mixing with line-by-line spectral phase control.

Bulk implementations of the QFP have enabled arbitrary single-qubit rotations^41^, high-dimensional discrete Fourier transforms^42^, Bell-state analysis^43^, and controlled-NOT gates^44^. Moving toward integrated platforms, electro-optic frequency beam splitters have been implemented in thin-film lithium niobate using coupled resonators^45^, alongside frequency shifting and bandwidth compression of heralded single photons^46^. In parallel, integrated line-by-line pulse shapers based on silicon microresonators have achieved spectral resolutions down to 3 GHz^47^ and have been used to control time correlations of entangled photon pairs^48^. Fast electro-optic modulation in thin-film lithium niobate has also been heterogeneously integrated with silicon microresonators to generate photon pairs with tunable spectral purity^49^. Coherent FB manipulation has also been shown through all-optical approaches^32,50,51^ and very recently theoretically proposed using acousto-optic modulation and multi-modal phase matching in waveguides^28^.

Despite these advances, frequency-bin quantum information processing remains less mature than some other DoFs. A major limitation towards scalability has been the lack of a fully integrated platform that simultaneously supports nonclassical light generation, high-speed coherent frequency manipulation, and programmable spectral control.

In this work, we introduce a fully integrated QFP that unifies all three capabilities on the silicon photonic platform. Our processor integrates a microresonator-based biphoton quantum frequency comb source with controllable linewidth, a pump-rejection filter, two high-speed phase modulators, and a four-channel microring-resonator-based waveshaper. We demonstrate tunable frequency beamsplitters using single-tone RF modulation, achieving success probabilities exceeding 94% and fidelities above 99.9%. Following Ref. ^41^, we implement general single-qubit gates through RF-delay control and programmable spectral phases. Finally, we demonstrate full quantum functionality by generating photon pairs entangled across multiple frequency modes via spontaneous four-wave mixing. High-dimensional states are coherently manipulated to show both ballistic and strongly confined energy transport in two-photon quantum walks^35^. Moreover, two-qubit Bell states are carved from the high-dimensional quantum microcomb, and we implement on-chip frequency-bin quantum state tomography (QST) with a fidelity of 95.7(3)%.

## Results

### Description of the programmable frequency processor

**Fig. 1:**
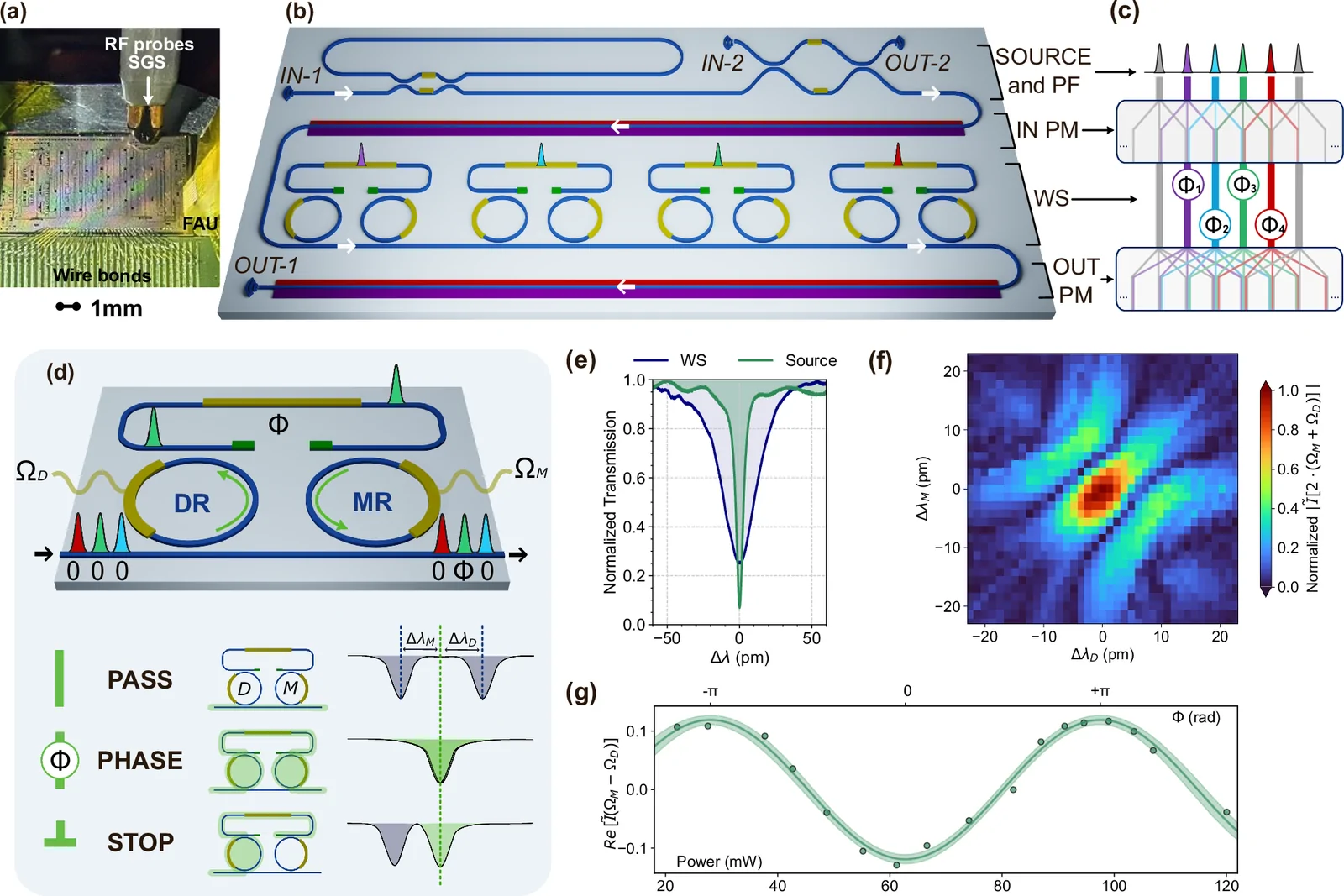
Integrated quantum frequency processor. **a** Photograph of the chip, indicating the fiber-array unit (FAU), the PCB wire-bonded to the on-chip heater pads (wire bonds), and the RF probes in the signal-ground-signal (SGS) configuration. **b** Schematic of the chip design. SOURCE: microresonator-based photon-pair source, PF: Mach-Zehnder-based pump-rejection filter, IN PM: input phase modulator, WS: four-channel line-by-line waveshaper, OUT PM: output fast phase modulator. Waveguides are shown in blue, heaters in yellow, Germanium absorbers in green, p-n junction-based modulators in red-purple. The pump (calibration) light is injected through the IN-1(IN-2) port, while photon pairs (filtered pump) are extracted from the OUT-1 (OUT-2) port. **c** Schematic of the processor operation. The generated input frequency bins first undergo mode mixing in the IN PM, then acquire programmable spectral phases ${\{{\Phi }_{j}\}}_{j=1}^{4}$ imparted by the WS, and are finally mixed again by the OUT PM. **d** Top: WS-unit working principle. The DEMUX ring (DR) drops a selected frequency bin (green), which is phase-shifted by *Φ* and subsequently multiplexed back into the common bus waveguide by the MUX ring (MR). DC-voltages and dither tones *Ω*_*D*(*M*)_ are applied to the rings' heaters for wavelength tuning and calibration purposes. Bottom: programmable channel operations, each associated with different light paths and relative resonance detuning *Δλ*_*D*(*R*)_ of the DR(MR) ring with respect to the target channel wavelength (dashed green line). **e** Measured spectral response of a WS-unit at *Φ* = *π* and of one resonance of the source as a function of the wavelength detuning *Δλ* from the channel wavelength. **f** Calibration of a WS-unit ring resonance wavelengths; measured amplitude of the Fourier transform $\tilde{I}$ at frequency 2(*Ω*_*M*_ + *Ω*_*D*_) of the time-dependent signal intensity *I*(*t*) as a function of *Δλ*_*D*(*R*)_. **g** Calibration of a WS-unit phase *Φ*; measured real part of the Fourier transform at frequency *Ω*_*M*_ − *Ω*_*D*_ as a function of the heater power *P*. The data are fit with the sinusoidal relation $\sim \sin \left(\frac{2\pi P}{{P}_{2\pi }}+{\Phi }_{0}\right)$ (the solid line shows the best fit for *P*_2*π*_ = (69 ± 1) mW, giving ~6^∘^ phase accuracy; the shaded region indicates the fit’s 95% confidence bounds). Both calibration procedures are detailed in the “Methods” section.

The silicon photonic frequency processor, reproduced in Fig. 1b, is fabricated within a multi-project wafer run on the 300 mm Si300 nm photonics platform at STMicroelectronics^52^ (more details on the waveguide and component geometries are provided in the “Methods” section). The chip area occupied by the circuit is approximately 4 × 7 mm^2^ and integrates all the basic functional building blocks required to implement a QFP. The optical circuit is reconfigured by on-chip heater elements, which are wire-bonded to an external printed circuit board (see Fig. 1a) and controlled by driving electronics. The whole sample is thermally stabilized using a Peltier cell.

Following Fig.  1b, a pump laser is fiber-coupled to the IN-1 port of the chip by using grating couplers, where it excites a microresonator-based photon-pair source designed to emit a BPFC by spontaneous four-wave mixing with a free spectral range (FSR) of ≃15.34 GHz. The resonator has a racetrack geometry and is tuned into the critical coupling regime by using an interferometric coupler. After the source, the pump is attenuated by means of an asymmetric Mach-Zehnder interferometer (MZI), acting as a pump rejection filter (PF) designed to avoid spurious photon-pair generation in the rest of the circuit. Additional off-chip filtering stages were implemented to perform coincidence measurements. The BPFC is then directed to the three-element QFP which, akin to the architecture implemented in Ref. ^41^, consists of a line-by-line waveshaper (WS) sandwiched between an input and an output fast phase modulator (IN PM and OUT PM). The intuition behind the working principle of the QFP is sketched in Fig. 1c. Each FB at the input is coherently mixed with its neighbors by the IN PM, then independent spectral phases are applied to each bin before a second mixing process occurs at the OUT PM. The phase profile applied by the WS, together with the relative phase and amplitude of the RF signals driving the PMs, determines how the frequency bins interfere. The process can be understood as a frequency-domain generalization of the MZI meshes based on beamsplitter networks and phase shifters used for reconfiguring the optical paths^53^, with the important subtlety that the mode mixing operated by the PMs is intrinsically high-dimensional. Specifically, mode mixing is implemented on chip by p-n traveling-wave carrier depletion modulators, each driven by a single tone RF signal whose frequency matches the FSR of the BPFC (see “Methods” section for fabrication and performance details).

The line-by-line WS consists of four cascaded pairs (WS-units) of add-drop microring resonators (MRR). The units can be operated in three different settings, as illustrated in Fig. 1d. In the standard PHASE configuration, the unit targets a specific channel wavelength and is used to control the phase of the corresponding frequency bin. The DEMUX ring of each pair (DR in Fig. 1d) drops a specific wavelength, which is routed towards the add-port of the second resonator after experiencing a programmable phase-shift *Φ*. The MUX ring (MR in Fig. 1d) adds back the dropped wavelength into the common bus waveguide, thus enabling independent phase control of the target comb line (Fig. 1c). To ensure a flat channel response across the BPFC modes, each ring has been designed to have a quality factor (Q) of about 5.2 × 10^4^, which is approximately six times lower than the loaded Q of the source (see “Methods” section for further details). A comparison between the WS-unit transmission spectrum and a resonance of the source is reported in Fig. 1e, while a typical spectrum of a single WS’s MRR is reported in Supplementary Fig. 2. In the PASS configuration, both rings are detuned from the channel wavelength and the FB is transmitted without any amplitude attenuation. In the STOP configuration, either the first or the second ring resonance is aligned to the central channel wavelength, directing the light towards a Germanium-based absorber that blocks its propagation. The calibration of each PHASE channel is done in two steps. First, the resonances of the two MRRs are aligned to the central channel wavelength. Then, the phase *Φ* is set to the target value. These steps are implemented using feedback signals derived from the amplitudes of higher-order harmonics in the transmitted intensity, which are generated by small dither tones applied to the voltages of the heaters of the two rings (see Fig. 1f, g). The procedure (detailed in the “Methods” section) allows optimizing the WS settings despite the presence of thermal crosstalk between the components.

### Frequency beamsplitters and single-qubit gate synthesis

**Fig. 2:**
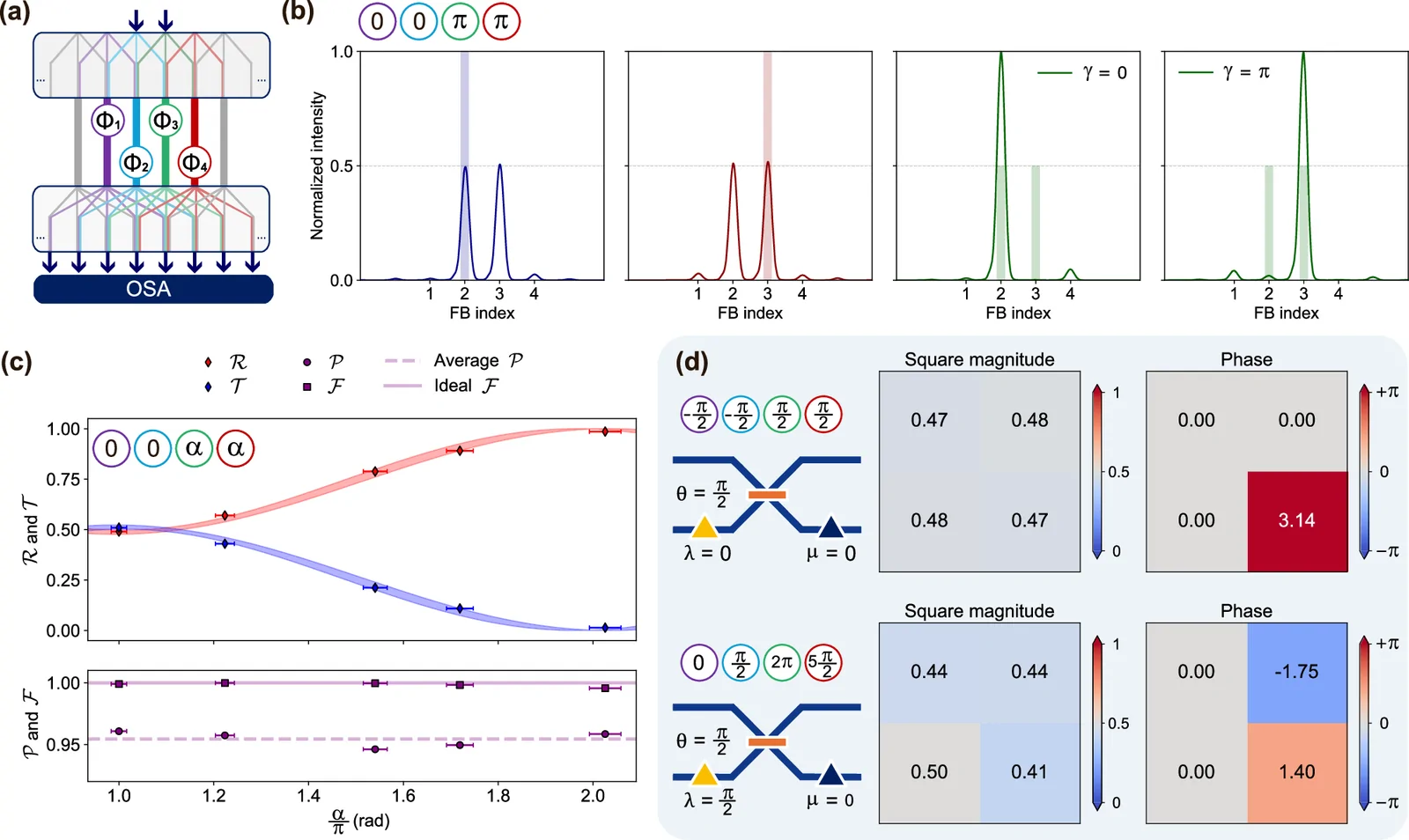
Implementation of frequency beamsplitters (*R*_*y*_ rotations) with tunable splitting ratios and *R*_*z*_ rotations. **a** Schematic of the processor configuration used to implement frequency beamsplitters with a tunable splitting ratio. An external probe laser is injected either into FB 2 or FB 3, or into a coherent superposition of the two. After the IN PM, programmable spectral phases ${\{\Phi \}}_{j=1}^{4}$ are applied to four modes before they enter the OUT PM. The spectral response at the output of the processor is recorded by an optical spectrum analyzer (OSA). **b** Measured output spectra when the processor is programmed to realize a 50/50 beamsplitter. The four spectral phases ${\{\Phi \}}_{j=1}^{4}$ applied by the WS are shown in the top inset of the leftmost panel. From left to right: input (shaded vertical line) in FB 2, input in FB 3, input in a coherent superposition of FBs 2 and 3 with relative phase *γ* = 0, and with a relative phase *γ* = *π*. **c** Top: tunable beamsplitter reflectivity ${{{\mathcal{R}}}}$ and transmittivity ${{{\mathcal{T}}}}$ as a function of the relative phase *α* applied between the FBs 2 and 3. The shaded regions represent the 95% confidence bounds predicted by the best fit that uses Supplementary Eq. (3), where the modulation index *δ* has been used as a free parameter (best fit value (0.843 ± 0.006) rad). Bottom: success probability ${{{\mathcal{P}}}}$ of the transformation and fidelity ${{{\mathcal{F}}}}$ with respect to the target operation. Errors on *α* are derived from the power-to-phase calibration of *Φ*(*P*) shown in Fig. 1g. The y-error bars are shown and are included within the marker size. **d** Top: reconstructed scattering matrix (squared magnitude and phase) of the 50/50 beamsplitter, corresponding to the operation *R*_*y*_(*θ* = *π*/2). The equivalent transformation, illustrated using dual-rail encoding, is sketched to the left of the matrices. The spectral phases applied to the four FBs are indicated inside the colored circles (see correspondence with panel (**a**)). Bottom: reconstructed scattering matrix of the transformation *R*_*y*_(*θ* = *π*/2)*R*_*z*_(*λ* = *π*/2).

We first use the processor to implement a fundamental building block for coherent frequency control: a deterministic beamsplitter between two frequency modes. This operation is nontrivial to implement in the frequency domain and cannot be achieved with a unit success probability–defined as the fraction of light remaining confined to the two input modes after the beamsplitter operation and normalized by the device insertion loss–using phase modulators alone, as they induce unwanted scattering of light into FBs outside the two modes of interest. However, as shown in Ref. ^41^, a three-element QFP, such as the one implemented on our chip and schematically depicted in Fig. 2a, can perform this operation almost deterministically.

To realize this operation, we require the two PMs to be driven by RF waveforms with a relative phase shift of *π*. When no spectral phase is applied by the WS, the frequency bins are scrambled by the IN PM and recombined out of phase at the OUT PM, effectively yielding the identity operation. By contrast, introducing a relative phase *α* between the frequency bins involved in the beamsplitter operation breaks the destructive interference at the OUT PM, enabling tunable splitting ratios. Crucially, destructive interference is simultaneously preserved for all non-targeted modes, thereby suppressing unwanted scattering (see Supplementary Note 1 for mathematical details).

We characterize the beamsplitter operation using two target frequency bins separated by *Δν* = 13.25 GHz and centered at a wavelength of 1547 nm. The input probe light is prepared off-chip using an electro-optic frequency comb filtered by a programmable waveshaper that selects and equalizes the amplitudes of the input modes (see “Methods” section for the detailed off-chip setup). For this characterization, the photon-pair source is bypassed by injecting light through the IN-2 port of the chip (Fig. 1b). The IN and OUT PMs are driven by a single RF tone at 13.25 GHz with a relative *π* phase shift and a modulation depth of *δ* ≃ 0.82 rad^54^. Using the on-chip WS, we independently control the phases ${\{{\Phi }_{j}\}}_{j=1}^{4}$ of the four channels, labeled 1 through 4 in Fig. 2a. The beamsplitter operation is implemented between the inner modes 2 and 3 by setting *Φ*_1_ − *Φ*_2_ = *Φ*_3_ − *Φ*_4_ = 0, and *Φ*_3_ − *Φ*_2_ = *α*. We initially focus on a 50/50 operation, which is realized by setting *α* = *π* (see Supplementary Note 1 and Ref. ^54^). In this condition, when light is separately injected into frequency bins 2 and 3, it is found to be equally split between the two output modes, as shown in Fig. 2b. To assess coherent operation, we prepare an equal superposition of the two modes at the input, with a relative phase *γ* that is varied using the external phase modulator generating the input electro-optic comb. Meanwhile, the output spectra are monitored with an optical spectrum analyzer. As shown in Fig. 2b, the output light can be fully routed to bin 2 or bin 3 by setting *γ* = 0 and *γ* = *π*, respectively, demonstrating the coherent mode mixing of the frequency beamsplitter.

We then vary *α* from *π* to 2*π* to tune its reflectivity ${{{\mathcal{R}}}}(\alpha )$ and transmissivity ${{{\mathcal{T}}}}(\alpha )$. Theoretically, ${{{\mathcal{R}}}}$ can be tuned from a minimum of 0.4978 by setting *α* = *π* to a maximum of 1 when *α* = 2*π*, while ${{{\mathcal{T}}}}$ varies from a maximum of 0.4781 for *α* = *π* to zero when *α* = 2*π*, following a cosinusoidal dependence (see Supplementary Eq. (4)). The measured ${{{\mathcal{R}}}}(\alpha )$ and ${{{\mathcal{T}}}}(\alpha )$, shown in Fig. 2c, are in good agreement with theory. Crucially, the success probability remains above 94% over the entire range *α* ∈ [*π*, 2*π*], well above the ~66% upper bound achievable with a single phase modulator.

The high success probability is further evidenced by the spectra shown in Fig. 2b, where the light intensity scattered into modes outside the target bins (2 and 3) is consistently at least an order of magnitude lower than that within the target modes.

We use the four spectra shown in Fig. 2b to reconstruct the complex 2 × 2 scattering matrix of the 50/50 beamsplitter^41^, shown in Fig. 2d. More details on the procedure used are reported in Supplementary Note 2. The fidelity, defined as the Frobenius overlap with the ideal target transformation, is ${{{\mathcal{F}}}}=0.999(1)$.

Having validated the beamsplitter transformation for different splitting ratios, which, within a quantum mechanical picture, can be described by the parametrized rotation *R*_*y*_(*θ*) = *e*^*iθY*^ (with *Y* denoting the corresponding Pauli operator) on the Bloch sphere, we then synthesize more general single-qubit gates. An arbitrary unitary operation on two modes would require two additional rotations, *R*_*z*_(*λ*) = *e*^*iλZ*^ and *R*_*z*_(*μ*) = *e*^*iμZ*^ (with *Z* denoting the corresponding Pauli operator), applied before and after *R*_*y*_(*θ*). Without loss of generality, we focus on the specific setting *θ* = *π*/2 (50/50 splitting ratio), *λ* = *π*/2, and *μ* = 0, but arbitrary rotation angles can be similarly implemented.

As shown in Ref. ^55^, the operation *R*_*y*_(*θ*)*R*_*z*_(*λ*) can be realized by starting from the processor settings that implement *R*_*y*_(*θ*) and then adding a phase shift −*λ* to the RF signal driving the IN PM, together with a linear spectral phase ramp applied to the WS, i.e. *Φ*_*k*_ ↦ *Φ*_*k*_ + *kλ* (similarly, the final rotation *R*_*z*_(*μ*) could be implemented by adding a phase shift −*μ* to the OUT PM and by updating the linear phase ramp as *Φ*_*k*_ ↦ *Φ*_*k*_ + *k*(*λ* + *μ*)). We applied this procedure and reconstructed the complex scattering matrix, which is shown in Fig. 2d (bottom panel). The fidelity of this matrix with respect to the target is 0.993(1), and the operation is performed with a success probability of 0.90(1).

These results demonstrate that, through accurate phase control over four comb lines, the device performs reproducible and high-fidelity single-qubit gates without significant population of frequency bins outside the computational subspace. Although the success probability could be marginally improved by controlling additional side channels with the WS, such gains would come at the cost of increased system complexity^41^. As a final remark, while the *R*_*y*_(*θ*) rotation shown in Fig. 2c is demonstrated over the range *θ* ∈ [0, *π*/2], corresponding to *α* ∈ [*π*, 2*π*], it could in principle be extended to the full range *θ* ∈ [0, *π*] by further increasing the modulation depth *δ*. However, as discussed in Ref. ^55^, larger values of *δ* necessarily reduce the success probability unless additional WS channels and RF tones are employed, which are beyond the capabilities of the processor in its current implementation.

### Tunable quantum walks and quantum state tomography

**Fig. 3:**
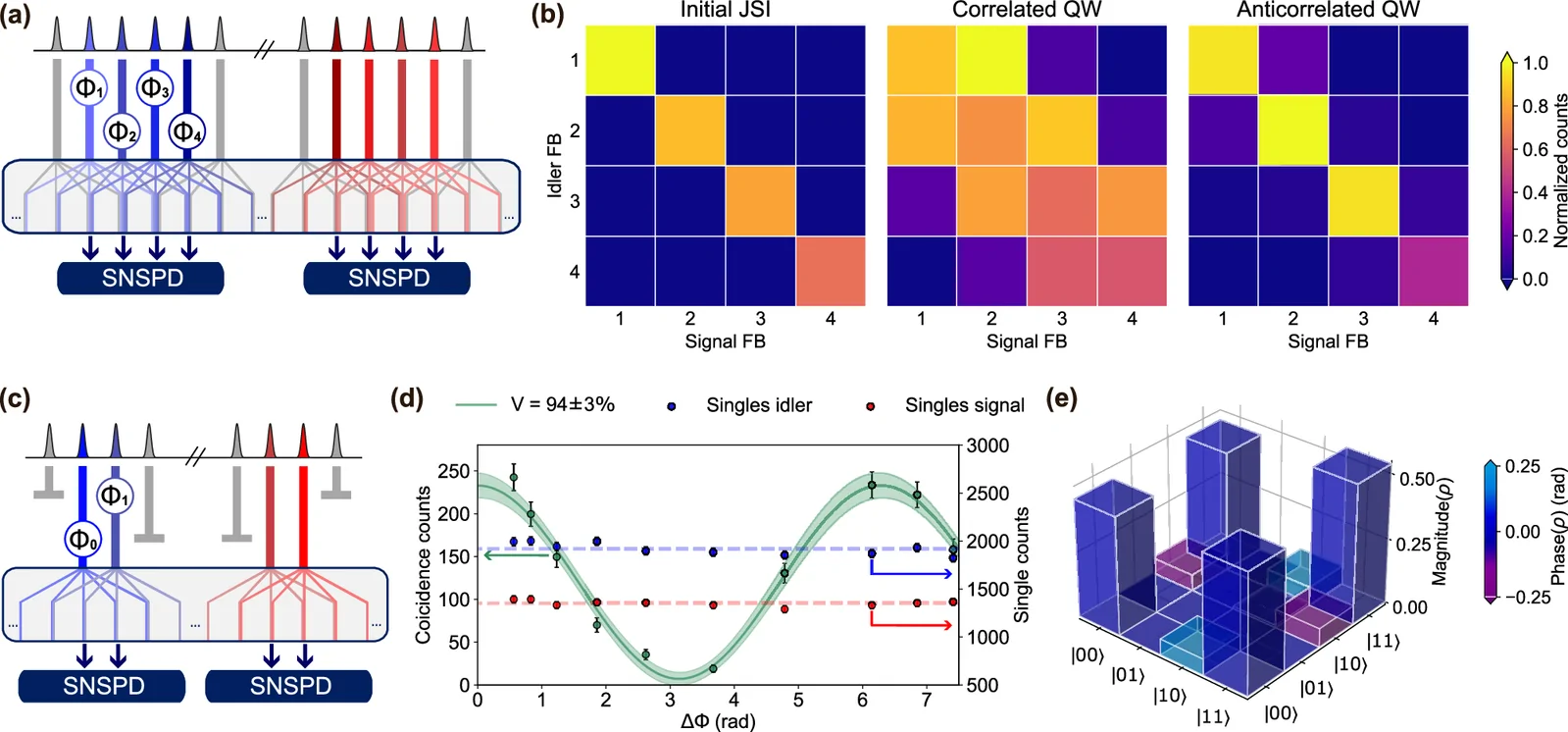
On-chip coherent manipulation of high-dimensional frequency-bin entangled states and quantum state tomography. **a** Schematic of the processor configuration used to implement high-dimensional quantum walks. A programmable spectral phase is applied to four idler (blue) modes, labeled from 1 to 4. Signal and idler modes are independently mixed at the OUT PM. Frequency-resolved signal-idler coincidence events are recorded off-chip using superconducting nanowire single photon detectors (SNSPDs). **b** JSIs in a restricted 4 × 4 subspace in three different processor configurations. From left to right: the initial JSI of the source, after the correlated quantum walk, and after the anticorrelated quantum walk. The recorded coincidence counts are reported here normalized to the maximum of each subspace. **c** Processor configuration used to implement on-chip two-qubit quantum state analysis. Two signal and two idler comb lines are isolated by guard-band modes. Spectral phases are applied to the idler FBs to control the measurement projectors, while the OUT PM enables projections onto superposition bases. **d** On-chip Bell state analysis. The measured coincidences (green) and single counts (blue and red) are reported as a function of the relative phase *ΔΦ* = *Φ*_1_ − *Φ*_0_ introduced between the FBs by two WS channels. The green curve (solid line) is a sinusoidal fit of the data, where 95% confidence bounds are shown as shaded regions. **e** Reconstructed magnitude (height of the bars) and phase (color of the bars) of the density matrix through quantum state tomography of the two-qubit state, which closely approximates the maximally entangled Bell state $\left\vert {\Phi }^{+}\right\rangle$.

We now demonstrate the capability of the integrated QFP to manipulate the BPFC generated by the on-chip source. To this end, the microresonator is resonantly excited with a pump power of 2.5 mW, generating signal-idler photon pairs with an on-chip rate of ~1.0 MHz and a coincidence-to-accidental ratio (CAR) of 55 ± 12 for each resonance pair (see “Methods” section for the detailed experimental setup). In what follows, we will restrict the frequency-resolved coincidence measurements to the 4 × 4 subspace spanned by the four idler frequency bins passing the WS channels (labeled 1–4 in Fig. 3a) and their energy-correlated signal bins, due to the limited bandwidth (BW ~ 80 GHz) of the filters implemented off-chip for idler-signal separation and pump suppression. Figure 3b (Initial JSI panel) shows the measured joint spectral intensity (JSI) over this sixteen-dimensional space corresponding to the state generate immediately after the source, i.e., $\left\vert {\Psi } \right\rangle={\sum}_{n} {\beta}_{n} \left\vert {nn}\right\rangle_{si}$, where ${\beta}_{n}$ are complex coefficients. No background noise from photon pairs generated off-resonance is observed, confirming efficient suppression by the pump-rejection filter.

To demonstrate coherent manipulation of this high-dimensional state, we apply a single RF tone at 15.34 GHz, matching the FSR of the resonator, with a modulation depth of *δ* ≃ 0.82 rad to the OUT PM, while keeping the IN PM off and the four WS channels in the PASS configuration. The applied RF signal coherently mixes FBs within both the signal and idler subspaces, effectively inducing a frequency-domain quantum walk whose depth increases with *δ*. As discussed in Ref. ^35^, the efficiency of energy exchange between the modulator and the photon pair depends critically on the relative phase $\Delta={{{\rm{Arg}}}}({\beta }_{l+1})-{{{\rm{Arg}}}}({\beta }_{l})$ between adjacent frequency bins prior to the walk: is maximized for *Δ* = 0 and minimized for *Δ* = *π*, corresponding to correlated (ballistic) and anticorrelated (strongly confined) quantum walks, respectively. The biphotons generated by the MRR naturally exhibit *Δ* ≈ 0 (see numerical simulations in Supplementary Note 6), and therefore, by turning on the OUT PM, they undergo a correlated walk, with frequency correlations spreading away from the main diagonal as shown in Fig. 3b (correlated QW panel). We then convert the walk from correlated to anticorrelated by applying a relative phase *Δ *= *π* between adjacent idler frequency bins using the integrated WS. The resulting JSI, shown in Fig. 3b (Anticorrelated QW panel), clearly demonstrates an increased confinement of the biphoton energy along the main diagonal. The WS is essential for this operation because both photons propagate through the same PM. Routing the idler and signal photons through separate PMs would allow an equivalent phase shift to be introduced by delaying one of the RF drive signals; however, this approach would require additional modulators, RF lines, and spectral filtering stages, thereby increasing circuit complexity and footprint.

Finally, we exploit the independent control of signal and idler photons to perform on-chip quantum state tomography of two frequency-encoded qubits. The state is carved from the broadband BPFC by creating guard bands around two signal and two idler frequency bins, labeled 0 and 1 in Fig. 3c, yielding a state well approximated by the Bell state $\left\vert {\Phi }^{+}\right\rangle=\frac{1}{\sqrt{2}}({\left\vert 00\right\rangle }_{si}+{\left\vert 11\right\rangle }_{si})$. Guard bands are implemented using four MRRs of the WS (STOP configuration), providing approximately 12 ~ 15 dB suppression of the unwanted modes. The remaining four MRRs, forming two WS channels in the PHASE configuration, apply programmable spectral phases to the idler modes while leaving the signal modes unaffected (PASS configuration). With the OUT PM turned on and by performing a frequency-resolved coincidence measurement on ${\left\vert 00\right\rangle }_{si}$ as a function of the relative phase *ΔΦ* = *Φ*_1_ − *Φ*_0_ between the idler FBs, we observe the two-photon interference fringe reported in Fig. 3d. The visibility obtained from the fit (V) is 94(3)%, violating the Bell inequality by more than 7 standard deviations.

Furthermore, a set of projective measurements is realized to perform on-chip quantum state tomography by combining spectral phase control with frequency mixing via the OUT PM (the latter turned off for projections on the computational basis). The density matrix, reconstructed via maximum-likelihood estimation^29^, and employing threshold quantum state tomography methods^56^ to overcome the incompleteness of the projective measurement set (see Supplementary Note 3 for methodological details), is shown in Fig. 3e. The measured fidelity with respect to the target Bell state $\left\vert {\Phi }^{+}\right\rangle$ is 0.957(3), and the purity 0.938(5).

## Discussion

We demonstrated key elements required for frequency-bin quantum information processing, namely quantum state generation, coherent frequency mixing, and spectral phase control. This is enabled by the monolithic integration and the high device uniformity provided by the silicon photonic platform. We observed highly reproducible performance across all processor configurations, calibrated through electronic and thermal control parameters, demonstrating that complex linear transformations between frequency bins can be implemented all on-chip with high accuracy and precision. In addition, the strong third-order nonlinearity of silicon and tight optical mode confinement enable the co-integration of an on-chip photon-pair source, while fast carrier-depletion modulators provided the required frequency-mixing capability.

The waveshaper architecture proposed here, in contrast to recent integrated microring-based pulse shapers^47^, is intrinsically non-blocking, preserving all frequency bins on a single bus waveguide and enabling lossless pass-through operation for selected modes while coherently manipulating others. In perspective, the QFP architecture could address a larger number of modes by adding MRR pairs to the line-by-line WS, thereby enabling the implementation of parallelizable gates as demonstrated with bulk components in Ref. ^24^. Further scaling of the processor’s information capacity could be achieved by reducing the frequency bin spacing, which would require a reduction in the round-trip optical losses of the MRRs and adapting the source design accordingly, with ultralow-loss platforms such as silicon nitride representing a promising pathway toward this goal.

Universal transformations on *N* modes likely require ${{{\mathcal{O}}}}(N)$ WS-PM blocks (more precisely, in Ref. ^18^, it is shown that an upper bound on the number of components is 2*N* − 1, while determining the minimum number of elements remains an open problem and currently relies on numerical optimization). Therefore, integration is a necessary step toward the realization of large-scale QFP, and our work marks important progress in this direction. Equally important is the availability of a clear strategy to reduce the loss associated with each component. At present, the waveshaper contributes ~6 dB of loss and currently represents the main performance limitation of the architecture, although this value is consistent with similar integrated implementations^47^. However, this can be substantially mitigated by increasing the coupling coefficient between the MRRs and the bus waveguides, or by employing asymmetric couplers to improve the drop efficiency. Phase modulators based on carrier depletion suffer from intrinsic carrier absorption, which typically introduces an insertion loss of 0.5–1 dB for a 1-mm-long device^57^. Alternatives are actively being explored, including the integration of CMOS-compatible electro-optic polymers^58^ and the heterogeneous integration of thin-film lithium niobate on silicon^59^.

In principle, the current footprint of our processor would allow the manipulation of tens of modes. In practice, the losses associated with each WS-PM block would need to be significantly reduced to <1 dB/block to be competitive with state-of-the-art universal photonic processors based on spatial encoding^60^. However, we emphasize that one of the major strengths of frequency-bin manipulation via electro-optic modulation lies in the possibility of driving the modulators with multiple RF harmonics. This approach has already been demonstrated to be highly effective for implementing high-dimensional Fourier gates, achieving dimensions up to 10 using only a three-element quantum frequency processor^42^. Therefore, increasing the number of driving harmonics and the modulator bandwidth while maintaining a limited physical circuit depth represents a highly promising strategy for reducing the overall loss of the processor.

Equally compelling are alternative architectures that may reduce the loss and the resource cost of the QFP scheme. For example, a theoretically proposed approach employs *N* cascaded resonators, each driven by *N*-RF tones spaced by the resonator FSR, to implement arbitrary unitary transformations on *N* modes^61^. This strategy eliminates the need for a waveshaper, shifting the system complexity to RF waveform generation and mixing. Another promising approach exploits coupled resonators for frequency-domain beamsplitter operations^62^. Such devices can be arranged into a universal triangular architecture with only nearest-neighbor mode coupling^63^, an idea that has been partially explored in variational circuits for multimode squeezed-light decomposition^64^. This strategy is particularly appealing because it can be implemented using compact microring resonators with large FSRs and requires only a single RF tone matched to the frequency separation of the supermodes of the photonic molecule^62^.

Promoting our architecture to a fully monolithic platform for quantum information processing, free of external optical components, will require the integration of high-extinction-ratio pump filters, signal-idler demultiplexers, and ultimately single-photon detectors together with dedicated optical isolation and scattering-attenuation structures. All of these building blocks have already been demonstrated in silicon-based photonic platforms^65^. Near-term silicon-based frequency processors may therefore be well suited for protocols and applications that do not require ultra-low-loss operation. A notable example is decoy-state quantum key distribution (QKD), where silicon photonic transceivers based on time-bin or polarization encoding have already demonstrated excellent performance^66^, as well as related protocols such as coherent one-way QKD^67^ and measurement-device-independent QKD^68^. Equally attractive are frequency processors operating outside the single-photon regime, with applications including pulse shaping, neuromorphic computing, and complex-field spectrum analysis.

## Methods

### Detailed description of the processor layout

The silicon photonic chip is fabricated within a multi-project wafer using the PIC50 technology of STMicroelectronics. The waveguides are implemented in single-mode RIB geometry with a 300 nm-thick silicon core, a 150 nm partial etch, and widths of 450 nm (core) and 3050 nm (slab). The propagation losses are estimated to be ~1.2 dB/cm and contribute to 3.4 dB of loss in the processor. Light is coupled into and out of the chip through grating couplers, which have an insertion loss of 3.1 ± 0.5 dB at 1555 nm. The insertion loss of the whole chip, including the grating couplers, is 13.0 dB (from IN-1 to OUT-1, at 1555 nm, with all the MRRs’ resonances detuned from the probing wavelength).

The source consists of a ~5.1 mm-long resonator whose coupling coefficient to the bus waveguide can be tuned via thermal phase shifters. This is achieved using a symmetric MZI in a push-pull configuration. The experiments are performed close to the critical-coupling regime, corresponding to an average quality factor of 3 × 10^5^ over the resonances of interest and a measured brightness per resonance of 298 ± 1kHz/mW^2^. The source is followed by a pump rejection filter (PF in Fig. 1b) based on an asymmetric MZI with a free spectral range of 500 GHz. The filter provides approximately 30 dB of attenuation at the pump wavelength (1555.9 nm) and ~0.2 dB of insertion loss at the signal-idler wavelengths, which are located at approximately 1550.3 nm and 1561.6 nm, respectively. The filter offers additional input and output ports for calibration and testing (IN-2 and OUT-2 in Fig. 1b).

Four WS-units are included in the circuit, composed of a pair of nominally identical MRRs of FSR = 250 GHz, arranged as depicted in Fig. 1d. The channel wavelength of each WS-unit and the phases ${\{\Phi \}}_{l=1}^{4}$ are controlled through thermal phase shifters. In the PHASE configuration, the non-unit drop efficiency results in an insertion loss of 5 ~ 6 dB per channel, with a small dependence on the applied phase *Φ* (see the experimental spectra in Supplementary Fig. 2). In the PASS configuration, losses are negligible since photons are not coupled to the resonators. The reported loss values are consistent with similar integrated architectures^47^ and improvable by increasing the coupling coefficient of the MRRs.

The chip integrates two silicon carrier-depletion modulators, referred to as IN PM and OUT PM in Fig. 1b, based on 3 mm-long p-n junctions embedded in a deep-RIB waveguide geometry with a slab thickness of 50 nm and a core width of 360 nm, in which the doping regions are specifically engineered for high-speed operation. More details on the cross-section and fabrication process can be found in Ref. ^52,69^. The two modulators are integrated side by side, with light co-propagating in the same direction, enabling RF excitation using a single two-signal RF probe. A common traveling-wave electrode, designed in a signal-ground-signal (SGS) configuration, is integrated above the two junctions and features a line impedance of 50 Ω with load termination. Each PM is driven by an independent RF signal and is simultaneously biased at −2 V through a bias-tee to ensure reverse-bias operation. The estimated insertion loss of each modulator is approximately 1.5 dB at 1550 nm. The measured junction modulation efficiency is 6.45 ± 0.15^∘^ mm^−1^ V^−1^ in the DC regime; strategies to increase the modulation efficiency in the present photonic platform can be found in Ref. ^70^.

In the experimental demonstrations reported here, the modulators are driven by single-tone RF signals with electrical powers ≃27–30 dBm, corresponding to modulation depths in the range *δ* = 0.8–1.4 rad. These power levels are consistent with the measured electrical loss of the traveling-wave electrode at the operating frequencies (13–15 GHz), which is approximately 9 dB. The relative RF phase between the two PMs is calibrated by setting each WS-unit to the PASS configuration and injecting a continuous-wave laser at the processor IN-2, with both the input and output PMs driven at equal modulation depth. The RF phase of the output PM is then varied until the electro-optic comb generated by the input PM is mapped back onto the carrier frequency by the second PM. This condition occurs for a relative RF phase of *π*, which is then used for phase reference.

### Detailed description of the setup

Here we provide a detailed description of the off-chip setup, whose schematic is included in Supplementary Fig. 1 for brevity. A tunable CW pump laser is sent to an off-chip electro-optic phase modulator driven by an RF signal at the modulation frequency used in the experiment (13.25 or 15.34 GHz). The resulting electro-optic comb lines are then selected by configuring an off-chip waveshaper as a passband filter centered on the target input-bin frequency. After polarization optimization and, when required, suppression of residual broadband spectral components using a bandpass filter, the light is injected into either the IN-1 or IN-2 port shown in Fig. 1. The on-chip operations are reconfigured by tuning the DC bias applied to the thermal phase shifters using dedicated computer-controlled voltage drivers. During the calibration of a single WS-unit, dither tones are applied to its MRRs to track their resonance positions and induced phase shifts (see the next section for details of the calibration procedure).

By adjusting the thermal phase shifter of the PF, different on-chip wavelengths can be routed either toward the rest of the chip or directly coupled off-chip through the OUT-2 port. For gate synthesis and calibration of the WS channels, the pump laser is injected into IN-2, thereby bypassing the source, and routed toward the QFP. After frequency-bin manipulation by the PMs and the WS, the light is coupled off-chip and measured either with an OSA, for gate characterization, or with a photodiode, for WS channel calibration.

For the source generation rate characterization, the quantum walk and quantum state tomography demonstrations, the pump light is instead injected into IN-1, tuned into resonance with the source, and routed off-chip through OUT-2. The transmitted laser light is monitored with a photodiode, and the pump laser current is adjusted in an active feedback loop to maintain the laser wavelength locked to the center of the pump resonance. The generated BPFC, shaped by the sine-like transmission spectrum of the PF, is directed toward the rest of the chip (the QFP), collected from the OUT-1 port and directed to a frequency-resolved single photon detection setup. The signal photons are separated from the idler and the pump photons through a DWDM filter (bandwidth ~80 GHz, ~80 dB of adjacent channel isolation). A tunable bandpass filter is then used to separate the idler frequency modes from the pump light (bandwidth ~15 GHz, >60 dB of adjacent channel isolation). The individual frequency bins are discriminated with tunable narrowband filters based on fiber Bragg-gratings (bandwidth ~12 GHz) and finally sent to the SNSPDs.

### Calibration of the line-by-line waveshaper

The calibration of the WS follows two main steps.

#### Alignment of the ring resonances

In the first step, the resonances of the DR and MR rings in Fig. 1d are aligned to the central channel wavelength *λ*_ch_. This is achieved by applying two low-amplitude dither tones at frequencies *Ω*_*D*_ = 150 Hz and *Ω*_*M*_ = 250 Hz to the heaters of the DR and MR rings, respectively, thereby inducing a small modulation of their resonance wavelengths with amplitude *Λ*. The resulting time-dependent transmitted intensity *I*(*t*) is recorded using a photodiode, and its spectral content is extracted via a fast Fourier transform (FFT).

The strongly nonlinear spectral response of the WS channel around the probe wavelength generates multiple harmonics in the FFT spectrum. The complex amplitudes of these harmonics depend on the relative detunings $\Delta {\lambda }_{D(M)}={\bar{\lambda }}_{D(M)}-{\lambda }_{{{{\rm{ch}}}}}$ between the average resonance wavelengths of the DR(MR) rings, ${\bar{\lambda }}_{D(M)}$, and the channel wavelength *λ*_ch_, as well as on the phase *Φ* imparted by the WS channel.

Using numerical simulations based on standard scattering-matrix formalism (see Supplementary Note 5 for details)– in which the resonance wavelengths of the DR and MR rings are modulated as ${\lambda }_{D(M)}(t)={\bar{\lambda }}_{D(M)}+\Lambda \sin ({\Omega }_{D(M)}t)$ – we find that the magnitude of the harmonic at frequency 2(*Ω*_*D*_ + *Ω*_*M*_) is maximized when *Δλ*_*M*_ = *Δλ*_*D*_ = 0, independent of the value of *Φ*. This condition, therefore, provides a robust criterion for aligning the resonances of each ring pair within a WS channel.

More in detail, the calibration starts with an initial coarse alignment of the resonances of the microrings in each WS-unit near their target wavelengths (~5 min, using a continuously scanning laser). After this procedure, which is performed only once at initialization, a laser is injected at *λ*_ch_ through the IN-2 port and routed to the first WS-unit. A grid search over heater currents is implemented to maximize the amplitude of the 2(*Ω*_*D*_ + *Ω*_*M*_) harmonic. The wavelength span covered in this phase is *Δλ* ~ 40 nm and takes about 20 min. An example of such a scan is shown in Fig. 1f. At the end of the scan, the heater currents maximizing the harmonic amplitude are stored, and the procedure is sequentially executed for each of the four WS-units. This procedure is performed only once. Then, a final narrower grid search of all units (*Δλ* ~ 10 nm, ~3 min per unit) is performed to compensate for the residual thermal cross-talk.

#### Mapping of the heater power to the phase Φ

The second step consists of determining the relationship between the electrical power *P* applied to the heater located between the DR and MR of a WS-unit and the induced spectral phase *Φ*. Numerical simulations show that, when the resonances are aligned (*Δλ*_*D*(*M*)_ = 0), the real part of the harmonic component at frequency *Ω*_*M*_ − *Ω*_*D*_ follows the simple relation ${{{\rm{Re}}}}(\tilde{I}({\Omega }_{M}-{\Omega }_{D}))={\tilde{I}}_{0}\cos (\Phi+{\Phi }_{0})$, where *Φ*_0_ is a constant phase offset corresponding to zero applied electrical power. Accordingly, after aligning the ring resonances as described in the previous step, we sweep the electrical power *P* applied to the heater and record a full interference fringe in ${{{\rm{Re}}}}\left[\tilde{I}({\Omega }_{M}-{\Omega }_{D})\right]$. To compensate for the thermal cross-talk induced on the microrings in the WS-unit, the narrow grid search described in the previous step is repeated for every different phase setting. The measured fringe is then fitted using ${{{\rm{Re}}}}\left[\tilde{I}({\Omega }_{M}-{\Omega }_{D})\right]={\tilde{I}}_{0}\cos (2\pi P/{P}_{2\pi }+{\Phi }_{0})$, where *P*_2*π*_ and *Φ*_0_ are treated as free fitting parameters. An example of such a fringe is shown in Fig. 1g. This procedure completes the phase calibration, as it establishes the mapping between the applied electrical power *P* and the induced phase, i.e., *Φ*(*P*) = *Φ*_0_ + 2*πP*/*P*_2*π*_.

Both calibration steps can be parallelized across multiple wavelength channels. To this end, an electro-optic frequency comb with the desired channel spacing can be generated either off-chip or directly on-chip using the IN PM, and subsequently demultiplexed at the WS output to provide a distinct signal for each WS-unit. In this configuration, the same pair of dither tones can be applied to the rings associated with each channel without introducing crosstalk.

Alternatively, distinct pairs of dither tones can be assigned to different WS channels, thereby eliminating the need to demultiplex the output signal into separate wavelengths. In this case, however, the dither frequencies must be carefully chosen to ensure that different WS-units do not produce identical harmonics.

For the proof-of-principle demonstration reported here, multiplexed calibration was not exploited. Instead, one WS-unit at a time was dithered, sequentially calibrating the four WS channels and iterating the procedure to improve calibration precision. Due to thermal crosstalk arising from the power dissipated by the phase modulators, calibration was performed while operating the modulators at the RF driving power of the experiment.

## Supplementary information

Supplementary Information

Transparent Peer Review file

## Data Availability

The data supporting the findings of this study are available within the Article and its Supplementary Notes. Additional data are available from the corresponding authors upon request.
