# Supplementary material for: Fully integrated quantum frequency processor on a silicon chip
Source: Nat Commun. 2026 Sep 15;17:10818. doi: 10.1038/s41467-026-77287-5 (PMC13669352; doi:10.1038/s41467-026-77287-5)
Supplement: Supplementary file 1 [file 41467_2026_77287_MOESM1_ESM.pdf]

Fully integrated quantum frequency processor on a silicon chip
Supplementary Information

SUPPLEMENTARY NOTE 1 - IMPLEMENTING SINGLE QUBIT ROTATIONS

The most general unitary operation acting on a single qubit is described by the matrix

$$U(\theta, \lambda, \mu) = \begin{pmatrix} \cos\left(\frac{\theta}{2}\right) & e^{i\lambda}\sin\left(\frac{\theta}{2}\right) \\ e^{i\mu}\sin\left(\frac{\theta}{2}\right) & -e^{i(\lambda+\mu)}\cos\left(\frac{\theta}{2}\right) \end{pmatrix}, \tag{1}$$

and can be decomposed into three sequential rotations $R_z(\lambda)R_y(\theta)R_z(\mu)$, parametrized by the angles (θ, λ, μ) where $R_J(x) = e^{ixJ}$ and $J = \{Y, Z\}$ are the Pauli matrices. We initially focus on the case $\mu = \lambda = 0$. The input space of frequency bins is infinite-dimensional, but we restrict our attention to the 2×2 subspace describing the coherent mixing between two modes of interest, that we label 0 and 1 and are spaced by a frequency separation $\Delta\nu$. In its simplest implementation, a three element QFP has been shown to realize arbitrary single qubit rotations by properly engineering the modulation depths of the two modulators, the relative phase of the RF single tones, and the spectral phases imposed by the WS [1]. Specifically, if the relative phase between the PMs is set to π and their modulation depth is set equal to δ, the splitting ratio of the beasmplitter can be controlled by a single parameter α, which is the relative phase between the bins 0 and 1 applied by the WS. In this configuration, all the bins $j < 0$ and $j > 1$ outside the computational basis have a zero relative phase offset, and the 2×2 matrix V acting on the subspace of bins 0 and 1 writes [2]

$$V(\alpha) = \begin{pmatrix} \sqrt{\mathcal{R}(\alpha)} & \sqrt{\mathcal{T}(\alpha)} \\ \sqrt{\mathcal{T}(\alpha)} & -\sqrt{\mathcal{R}(\alpha)} \end{pmatrix}, \tag{2}$$

where the reflectivity $\mathcal{R}(\alpha)$ and transmittivity $\mathcal{T}(\alpha)$ are

$$\mathcal{T} = \left| \left(1 - e^{i\alpha}\right) \sum_{k=1}^{\infty} J_k(\delta) J_{k-1}(\delta) \right|^2,$$

$$\mathcal{R} = \left| J_0^2(\delta) + \left(1 + e^{i\alpha}\right) \frac{1 - J_0^2(\delta)}{2} \right|^2, \tag{3}$$

and $J_k(\delta)$ is the Bessel functions of the first kind of order k. Note that the matrix in Eq.(2) is in general not unitary, reflecting the fact that some light can be scattered outside the computational space. These expressions can be simplified by simple algebraic steps, yielding

$$\begin{aligned} \mathcal{R}(\alpha) &= J_0^4 + \left(\frac{1 - J_0^4}{2}\right)(1 + \cos\alpha) \\ \mathcal{T}(\alpha) &= \bar{J}(1 - \cos\alpha) \end{aligned} \tag{4}$$

where $\bar{J} = 2|\sum_{k=1}^{\infty} J_k(\delta)J_{k-1}(\delta)|$. The 50/50 configuration is realized by setting $\delta = 0.8169$, for which $\bar{J} = 0.239$ and $V \approx U(\theta = \frac{\pi}{2}, 0, 0) = R_y(\frac{\pi}{2})$ [2].

The construction of arbitrary gates requires two additional Z rotations, one before $R_y(\theta)$, parametrized by the angle λ, and the other after the Y rotation, parametrized by the angle μ. In Ref.[2] the authors found a simple rule that relates $U(\theta, 0, 0)$ to $U(\theta, \lambda, \mu)$: delay the RF signal of the first and second PM by $\tau_a = -\frac{\lambda}{\Delta\nu}$ and $\tau_b = -\frac{\mu}{\Delta\nu}$ respectively, and add the linear spectral phase ramp $\phi_k \mapsto \phi_k + k(\lambda + \mu)$ to each WS channel k. This is the procedure that we implemented in our work (see Figure 2(d) of the main text) to realize the operation $R_z(\frac{\pi}{2})R_y(\frac{\theta}{2})$ from an initial configuration that realizes $R_y(\frac{\theta}{2})$, i.e a 50/50 beamsplitter.

Two figures of merit are employed to verify the quality of the implemented transformations: the fidelity $\mathcal{F}$, which evaluates the overlap between the (experimentally reconstructed) matrix V with the target U_t, and the success probability $\mathcal{P}$, which quantifies the fraction of light intensity remaining in the two target modes with respect to the total spectral intensity. Following Ref.[3], they are calculated using the Frobenius norm

as:

$$\mathcal{P} = \frac{1}{2}\,\mathrm{Tr}\big(V^{\dagger}V\big)\,, \tag{5}$$

$$\mathcal{F} = \frac{1}{4\mathcal{P}}\,\left|\mathrm{Tr}\big(V^{\dagger}U_t\big)\right|^2\,, \tag{6}$$

Note that since we are interested in evaluating the suppression of light into the unwanted modes $\neq (0, 1)$, the success probability $\mathcal{P}$ is normalized by the insertion loss of the device in the main text.

SUPPLEMENTARY NOTE 2 - CHARACTERIZATION OF THE BEAMSPLITTER SCATTERING MATRIX

The procedure used to characterize the scattering matrix closely follows the approach described in Ref.[4] and implemented in the frequency-bin platform of Ref.[3]. According to Ref.[4], one first defines a reference frame in which all elements of the first row and first column of the scattering matrix are real. Physically, this corresponds to an overall rotation of the output measurement basis and to a specific choice of the relative phases between the modes at the input of the N-mode interferometer. In particular, this phase convention maximizes the intensity in the first output mode when light is injected pairwise into modes 1 and n, with $n = 1, \ldots, N$.

For the 2×2 unitary matrix U describing the beamsplitter transformation in Equation (1), this convention implies that the quantities to be determined are $|U_{11}|$, $|U_{12}|$, $|U_{21}|$, and U_{22}. The elements $|U_{11}|^2$ and $|U_{12}|^2$ are obtained by injecting light into mode 1 (frequency bin 2, according to the convention of Figure 2(a) in the main manuscript) and measuring the output intensities in modes 1 and 2 (frequency bin 3 in Figure 2(a) in the main text) using a spectrum analyzer. The measured intensities are normalized to the intensity in mode 1 at the input of the interferometer. Similarly, $|U_{21}|^2$ and $|U_{22}|^2$ are determined by injecting light into mode 2 and measuring the output intensities in modes 1 and 2.

The elements of the first row of U are required to be real. This condition is enforced by injecting a coherent superposition of modes 1 and 2 and varying their relative phase using an external electro-optic modulator (EOM) until the intensity in output mode 1 is maximized. The phase of U_{22} is then determined by recording the intensity in output mode 2. This choice of reference frame yields the scattering matrix shown in the upper panel of Figure 2(d) in the main manuscript.

Once the reference frame has been established, the scattering matrix shown in the lower panel of Figure 2(d) in the main text, corresponding to a different qubit rotation, is characterized using a similar procedure. In this case, because the reference frame has already been fixed, the elements of the second column are allowed to be complex. The phases of U_{12} and U_{22} are determined by injecting a coherent superposition of modes 1 and 2 into input mode 1 and input mode 2, respectively, scanning their relative phase with the external EOM, and extracting the phase offset of the interference fringe relative to the reference configuration.

SUPPLEMENTARY NOTE 3 - DENSITY MATRIX RECONSTRUCTION WITH ASYMMETRIC PROJECTOR SETS

In our current implementation, the limited number of waveshaper channels do not allow to perform arbitrary single-qubit projectors to the signal and the idler photons simultaneously. When the WS is tuned to the idler frequency, the set of projectors available for the signal photons is limited to (in polarization notation) $\langle H|$ (horizontal), $\langle V|$ (vertical), $\langle D|$ (diagonal), and $\langle A|$ (anti-diagonal). Consequently, the tomographic set is not informationally complete.

Yet, to reconstruct the density matrix of the on-chip generated Bell state $|\phi^+\rangle = \frac{1}{\sqrt{2}}(|HH\rangle + |VV\rangle)$ we can exploit the fact that its density matrix is sparse and therefore characterized by fewer than the usual 15 independent real parameters representing arbitrary two-qubit states. For such low-rank states, a resource-efficient approach for identifying the most informative measurement settings is *threshold* quantum state tomography [5]. In this method, one first determines the diagonal elements of the density matrix and, based on these outcomes together with a threshold parameter t, selects the set of projectors that provides information about the most relevant density-matrix elements above the noise level. In principle, this set of projectors may be

asymmetric; for example, it may include $\langle DR|$ but not $\langle RD|$. To give a concrete example that directly applies to our case, we consider the set of projectors that bring direct information on the real and imaginary parts of the density matrix elements (Eq.(11) of Ref.[5]), which can be represented in a compact matrix form as

$$\pi_2 = \begin{bmatrix} |HH\rangle & |HD\rangle + i|HR\rangle & |DH\rangle + i|RH\rangle & |DD\rangle + i|DR\rangle \\ 0 & |HV\rangle & |RR\rangle + i|RD\rangle & |DV\rangle + i|RV\rangle \\ 0 & 0 & |VH\rangle & |VD\rangle + i|VR\rangle \\ 0 & 0 & 0 & |VV\rangle \end{bmatrix}, \tag{7}$$

where the projectors are arranged as $\mathrm{Re}(\rho_{ij}) = \mathrm{Re}(\pi_{2(i,j)})\rho\mathrm{Re}(\pi_{2(i,j)})^\dagger$ and $\mathrm{Im}(\rho_{ij}) = \mathrm{Im}(\pi_{2(i,j)})\rho\mathrm{Im}(\pi_{2(i,j)})^\dagger$. For $\rho = |\phi^+\rangle\langle^+\phi|$, the geometric mean of the elements $\rho_{22} = \langle HV|\rho|HV\rangle$ and $\rho_{33} = \langle VH|\rho|VH\rangle$, i.e. $\sqrt{\rho_{22}\rho_{33}}$, which has been determined from the experimental data, lies below our imposed threshold of $t = 0.01$. According to Ref.[5], all the elements of ρ in rows and columns 2 and 3 must be then ideally set to zero and not measured. The remaining set of projectors in Equation (7) are then $\langle DD|$ and $\langle DR|$, which is asymmetric since it does not contain the projector $\langle RD|$. After this selection, standard maximum-likelihood techniques employing a general unbiased parametrization of the density matrix, such as the Cholesky decomposition, is used to minimize the discrepancy between the expected and experimentally measured outcomes. In our case, the low-rank structure of the target density matrix enabled its faithful reconstruction from an asymmetric and tomographically incomplete set of projectors.

SUPPLEMENTARY NOTE 4 - ADDITIONAL FIGURES

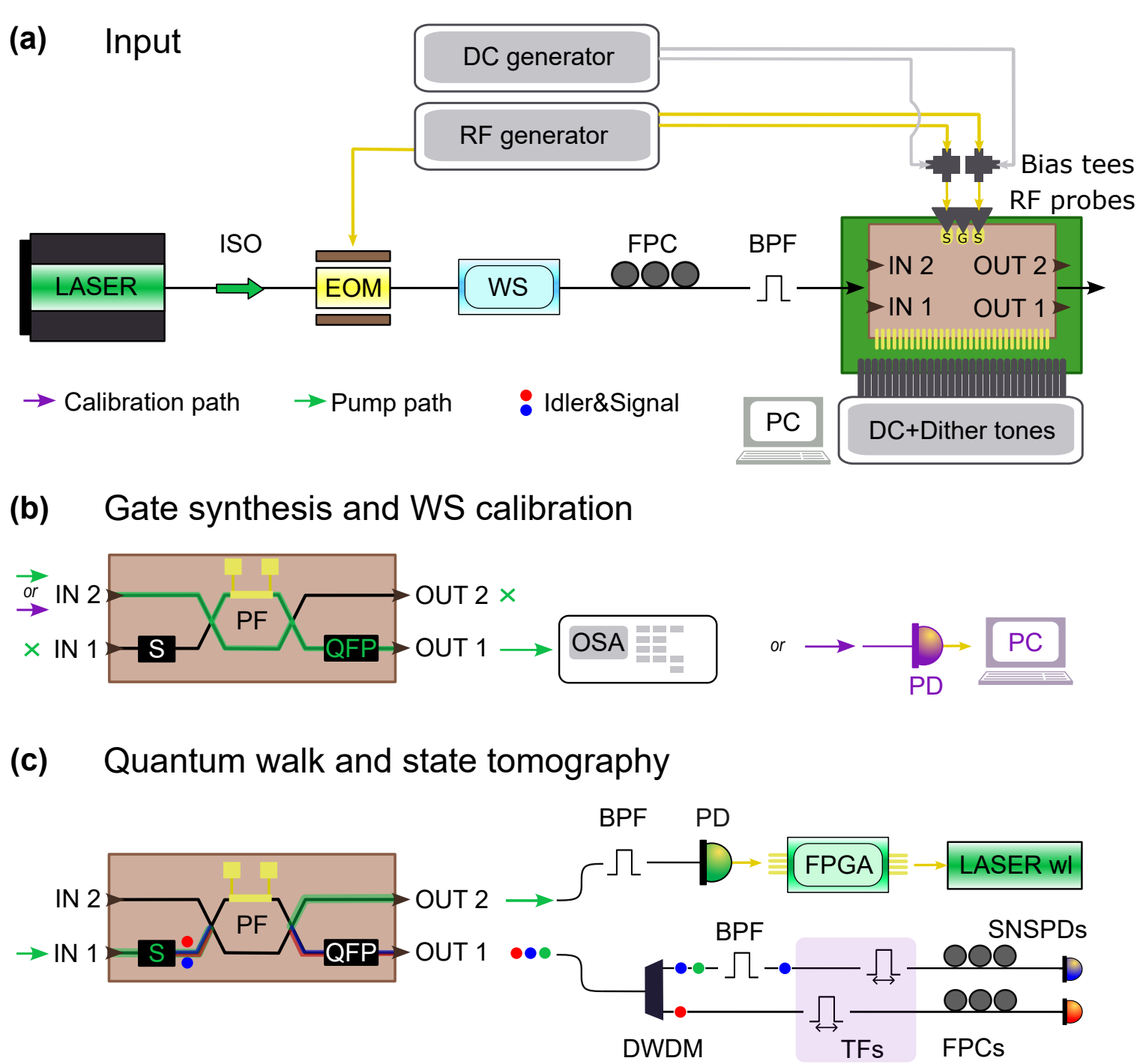

FIG. 1. **Detailed experimental setup.** (a) Setup at the input of the silicon chip, showing the main optical and electrical (DC bias, dither tones and RF signal) connections. (b) Configuration of the chip and setup used for gate synthesis and WS calibration (PHASE operation). (c) Configuration of the chip and setup used for quantum walks and quantum state tomography demonstrations. ISO: fiber isolator, EOM: electro-optic modulator, WS: bulk off-chip waveshaper, FPC: fiber polarization controller, BPF: band pass filter, S: Source, PF: pump filter, QFP: quantum frequency processor, OSA: optical spectra analyzer, PD: photodiode, DWDM: dense wavelength division multiplexing, TF: tunable narrowband pass-band filter, SNSPD: superconducting nanowire single photon detector.

SUPPLEMENTARY NOTE 5 - WAVESHAPER CHARACTERIZATION AND SIMULATION

In Supplementary Figure 2(a,b), we report the measured spectrum of a single WS MRR and the spectra of a WS unit for different applied phase shifts, Φ. All spectra are shown as a function of the wavelength detuning $\Delta\lambda$, with respect to the central channel wavelength.
In the WS unit, photons at the target channel wavelength are dropped by the two MRRs and experience the phase shift Φ in the waveguide section located between them. The alternative path, in which photons remain in the bus waveguide and pass through both resonators, is more than two orders of magnitude less probable owing to the relatively high through-port extinction ratio at resonance (>12 dB per MRR). At wavelengths outside the target channel, the amplitudes associated with the two possible paths become comparable, resulting in an interference effect that makes the transmitted intensity dependent on Φ. This feature is exploited during the calibration phase discussed in the main text.
We simulated the time-dependent transmitted intensity of the unit while applying two independent dither tones at frequencies Ω_D and Ω_M to the two resonators, respectively DR and MR, as performed during the WS calibration procedure described in the Methods section of the main text. The simulations consider a pair of identical symmetric add–drop MRRs arranged in the here introduced WS-unit geometry, with a radius of approximately $50\,\mu$m, an extracted power coupling coefficient of 0.023, a waveguide propagation loss of $1.2\,\mathrm{dB\ cm}^{-1}$, and a simulated effective index of the waveguide of $n_{\mathrm{eff}} \simeq 2.8$.
The real and imaginary parts of the Fourier components of the time-dependent optical signal are recorded for different values of the MUX resonance detuning $\Delta\lambda_M$ and DEMUX resonance detuning $\Delta\lambda_D$. Supplementary Figure 2(d) reports the results obtained for the harmonic at frequency $2(\Omega_D + \Omega_M)$ for four different channel phase shifts. While the overall magnitude pattern depends on the applied phase, its absolute maximum is located precisely at $\Delta\lambda_M = \Delta\lambda_D = 0$. In particular, the right panel corresponding to a phase shift of π reproduces the experimental two-dimensional map shown in the main text.
Supplementary Figure 2(c) shows the simulated real part of the harmonic at frequency $\Omega_M - \Omega_D$ as a function of the applied phase, calculated in the absence of detuning. The harmonic amplitude exhibits the expected $\cos(\Phi)$ dependence, which is used to map the heater thermal power to the corresponding induced phase shift, as described in detail in the Methods section. The corresponding experimental results are reported in the main text.

SUPPLEMENTARY NOTE 6 - QUANTUM WALK SIMULATION

The quantum walks shown in Figure 3(b) of the main text are also reported in Supplementary Figure 3 to enable direct comparison with the simulations. The state at the input of OUT PM is written as $|\Psi\rangle = \frac{1}{\mathcal{N}}\sum_{l=0}^{5}\beta_l|ll\rangle_{si}$, where $\mathcal{N}$ is a normalization constant. The four frequency bins labeled from 1 to 4 span the 4×4 Hilbert space of interest, within which frequency-resolved coincidence measurements are performed. The outer bins, labeled 0 and 5, also participate in the quantum walk but cannot be measured due to the limited bandwidth ($\sim 80\,\mathrm{GHz}$) of the off-chip filters used for pump suppression. Frequency bins with indices $l < 0$ or $l > 5$ are not included in the simulations owing to the relatively small modulation depth ($\delta 1 simeq 0.8\,\mathrm{rad}$) applied to the OUT PM, which confines frequency-bin scrambling primarily to nearest neighbors. The coefficients $|\beta_l|^2$ for $l = 1$–4 are extracted directly from the measured JSI of the source (Supplementary Figure 3(a)). The dominant source of inhomogeneity among the coefficients—visible as a decreasing intensity along the main diagonal in the initial JSI panel—is the pump-rejection filter, which imposes a sinusoidal envelope with a free spectral range of 500 GHz on the broadband frequency comb. This envelope is used to extrapolate the values of $|\beta_0|$ and $|\beta_5|$.
To extract the relative phases between the input frequency bins, we sent the state $|\Psi\rangle$ through the OUT PM with the modulation depth set to $\delta = 0.8\,\mathrm{rad}$, and measured the resulting JSI, shown in Supplementary Figure 3(b). We then employed a numerical optimization procedure (`scipy.minimize`) to maximize the overlap between the experimentally measured JSI and a simulated distribution. The quantum-walk dynamics were simulated following the method described in the Supplemental document of Ref.[6], which we do not reproduce here for brevity. The free parameters in the optimization are the relative phases $\mathrm{Arg}(\beta_l)$ for $l = 1 - 4$, with $\mathrm{Arg}(\beta_0) = 0$ used as a reference. All other parameters were fixed based on independent experimental calibration, including the modulation depth δ, the losses of each WS channel, and the applied spectral phases $\{\Phi_l\}_{l=1}^{4}$, which were all set to zero. We find that the phases $\mathrm{Arg}(\beta_l)$ maximizing the overlap between the simulated ($\mathrm{JSI_t}$) and the experimental ($\mathrm{JSI_e}$) JSIs are all distributed within ± 0.1 rad. The

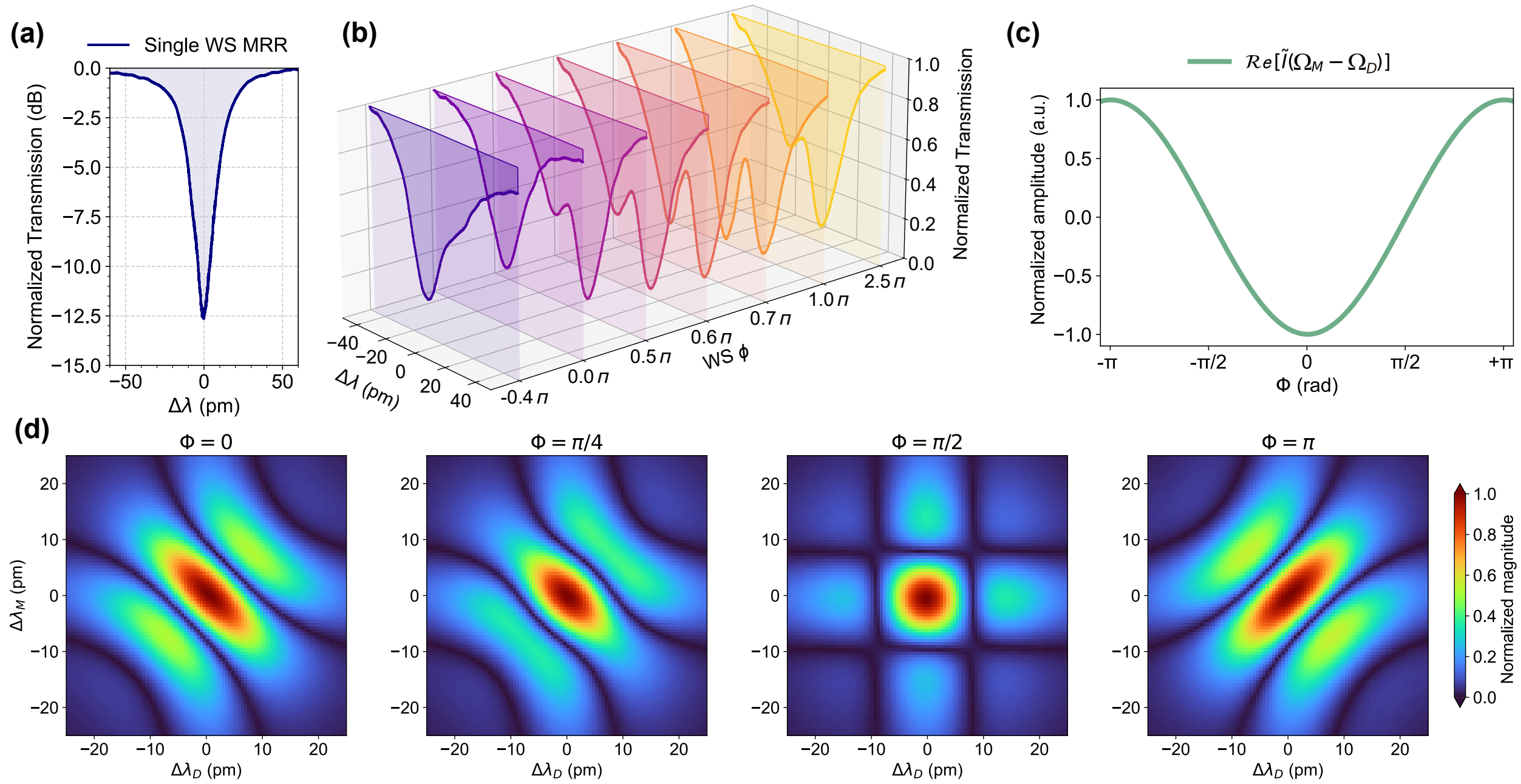

FIG. 2. **Additional WS characterization and simulation.** (a) Experimental spectrum of a WS's MRR. (b) Experimental spectra of a WS unit varying the applied phase shift Φ. (c) Simulated real part of the Fourier components at $\Omega_M - \Omega_D$ as a function of the phase-shift Φ applied by the WS-unit. The shown amplitude is normalized to the maximum of the curve. (d) Simulated magnitude of the Fourier component at $2(\Omega_D + \Omega_M)$ for different phases. For (c) and (d), the corresponding experimental results are reported in the main text, see Figure 1 (f) and (g), respectively.

simulation, shown in Supplementary Figure 3(b), has a fidelity $\mathcal{F} = \mathrm{Tr}([\mathrm{JSI_t}]^\dagger \mathrm{JSI_e})$ of $\mathcal{F} = 99.38(5)\%$. This indicates that the input frequency bins are mutually phase aligned within experimental and numerical uncertainty. Consequently, the generated photon pairs undergo a correlated quantum walk, characterized by frequency correlations spreading away from the main diagonal of the JSI [6].

To convert this behavior into an anti-correlated quantum walk, in which frequency correlations remain confined along the main diagonal, the input state must be transformed into $|\Psi\rangle_{\mathrm{ac}} = \sum_{l=0}^{5} |\beta_l| e^{il\pi} |ll\rangle_{si}$, thus a relative π phase shift must be introduced between consecutive frequency bins. This transformation is achieved using the four–channel WS. Specifically, a phase pattern $\Phi_{1-4} = [-0.5\pi, 0.5\pi, -0.5\pi, 0.5\pi]$ is applied on the four idler bins. Note that all photons of a single frequency mode are assumed to experience a uniform phase-shift across their narrow frequency band. This is justified by the >5 times higher bandwidth of the WS MRR with respect to the idler and signal resonances. The experimentally measured JSI corresponding to this configuration is shown in Supplementary Figure 3(c). After the OUT PM, the frequency correlations remain confined along the main diagonal, clearly indicating the transition to an anti-correlated quantum walk. The fidelity between the experimental JSI and the corresponding numerical simulation reported in Supplementary Figure 3(c) is $\mathcal{F} = 98.7(5)\%$, thus confirming that the above-mentioned assumption holds in the present case.

[1] H.-H. Lu, E. M. Simmerman, P. Lougovski, A. M. Weiner, and J. M. Lukens, Fully arbitrary control of frequency-bin qubits, Physical Review Letters **125**, 120503 (2020).
[2] H.-H. Lu, J. M. Lukens, N. A. Peters, B. P. Williams, A. M. Weiner, and P. Lougovski, Quantum interference and correlation control of frequency-bin qubits, Optica **5**, 1455 (2018).
[3] H.-H. Lu, J. M. Lukens, N. A. Peters, O. D. Odele, D. E. Leaird, A. M. Weiner, and P. Lougovski, Electro-optic frequency beam splitters and tritters for high-fidelity photonic quantum information processing, Physical review letters **120**, 030502 (2018).

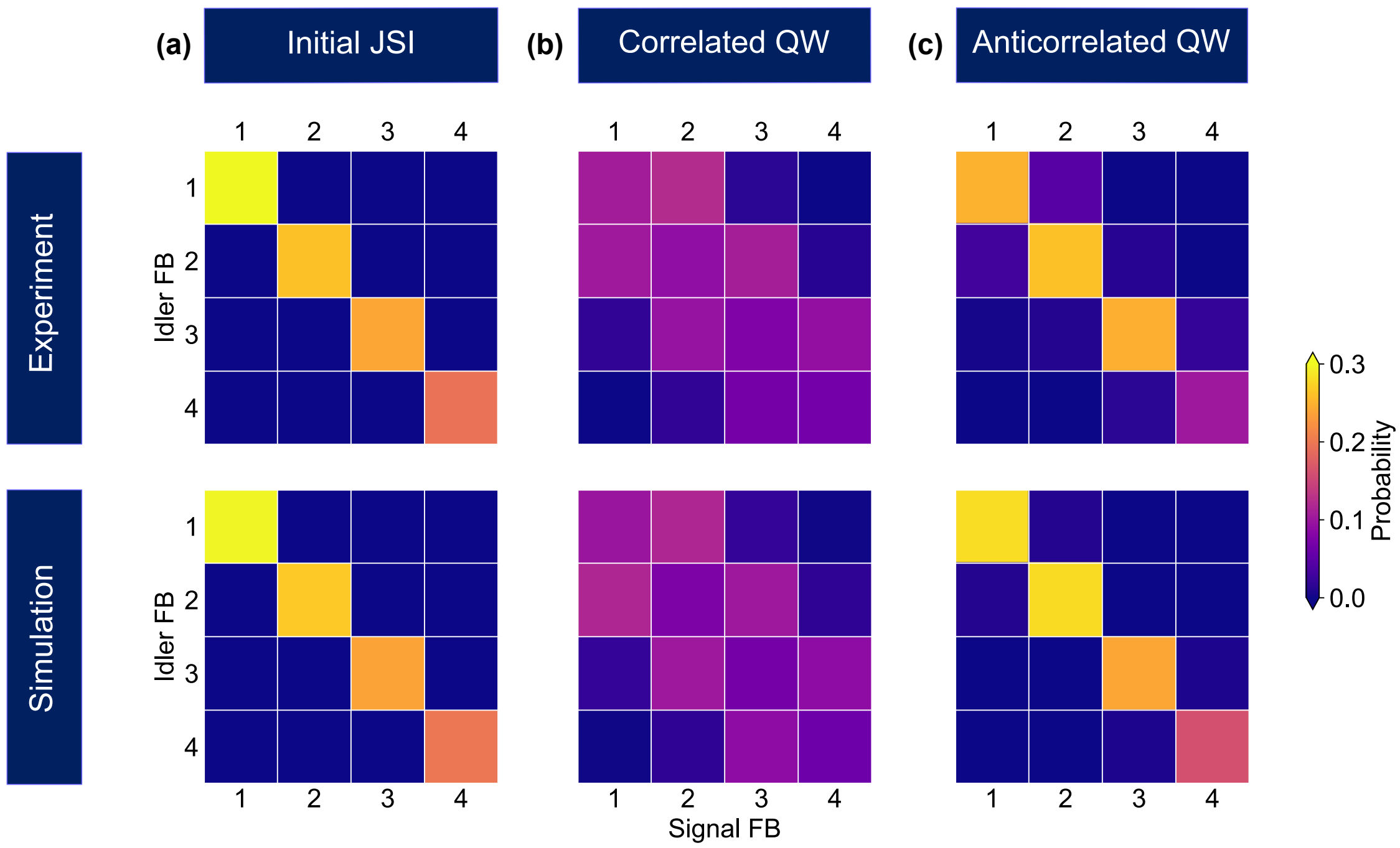

FIG. 3. **Experimental and simulated quantum walks.** (a) Initial JSI, coincidence pattern obtained directly after the source, keeping the PMs off and all the WS-units in the PASS configuration. It corresponds to the state $|\Psi\rangle$ described in the text. (b) Measured JSI after the OUT PM, showing a correlated quantum walk. (c) JSI measured after the OUT PM when the input state is $|\Psi\rangle_{\text{ac}}$, resulting in a anti-correlated quantum walk with low energy leakage outside the main diagonal. Top panels show the JSIs measured experimentally via frequency-resolved coincidence measurements. Bottom panels show the corresponding JSIs simulated using the model described in the Supplemental Document of Ref.[6]. Both experimental and simulated matrix entries have been normalized to the integral over the full 4×4 space.

[4] S. Rahimi-Keshari, M. A. Broome, R. Fickler, A. Fedrizzi, T. C. Ralph, and A. G. White, Direct characterization of linear-optical networks, Optics express **21**, 13450 (2013).

[5] D. Binosi, G. Garberoglio, D. Maragnano, M. Dapor, and M. Liscidini, A tailor-made quantum state tomography approach, APL Quantum **1** (2024).

[6] P. Imany, N. B. Lingaraju, M. S. Alshaykh, D. E. Leaird, and A. M. Weiner, Probing quantum walks through coherent control of high-dimensionally entangled photons, Science Advances **6**, eaba8066 (2020).
