# Supplementary material for: Fully integrated quantum frequency processor on a silicon chip
Source: Nat Commun. 2026 Sep 15;17:10818. doi: 10.1038/s41467-026-77287-5 (PMC13669352; doi:10.1038/s41467-026-77287-5)
Supplement: Supplementary file 2 [file 41467_2026_77287_MOESM2_ESM.pdf]

nature portfolio

Peer Review File

Fully integrated quantum frequency processor on a silicon chip

Corresponding Author: Ms Sara Congia

This file contains all reviewer reports in order by version, followed by all author rebuttals in order by version.

Version 0:

Reviewer comments:

Reviewer #1

(Remarks to the Author)
I co-reviewed this manuscript with one of the reviewers who provided the listed reports. This is part of the Nature Communications initiative to facilitate training in peer review and to provide appropriate recognition for Early Career Researchers who co-review manuscripts.

Reviewer #2

(Remarks to the Author)
In the manuscript titled ''Fully integrated quantum frequency processor on a silicon chip'' by Sara Congia, et al, the authors report a monolithic integration of a complete quantum frequency processor (QFP) on a silicon photonic platform. The authors successfully unify four critical functional blocks on a single chip and demonstrate high-fidelity frequency beamsplitting and on-chip quantum state tomography. The authors also provide a detailed and robust methodology for aligning ring resonances and mapping heater power to spectral phase, and the draft is easy to read. Overall, I recommend its publication in Nature Communications, but I still have a few questions.
1. The on-chip pump rejection filter provides around 30 dB attenuation. In some actual applications, 30 dB is not enough to isolate the single photons, for example in spontaneous four wave mixing. Did the authors use other filters (like off chip filters in this experiment) to obtain CAR of 55?
2. Were there any observed effects of thermal crosstalk from the high-speed phase modulators during precise thermal tuning of cavity resonances? If not, can the authors add some theoretical analysis?
3. In the "STOP" configuration, one ring is aligned to the channel wavelength to block light. What is the measured extinction ratio for a single frequency bin in this state?
4. In the tomography experiment, spectral phases were applied to the idler modes while signal modes remained in the "PASS" configuration. Does this asymmetric projection scheme introduce any systematic bias in the reconstructed density matrix?
7. How is the overall loss in each component of the whole chip? Which component has the highest loss and how does it influence the ultimate performance?
8. Based on current footprint and loss, what is the estimated maximum number of modes that could be realistically processed on a single wafer-scale chip?

Reviewer #3

(Remarks to the Author)
The work “Fully integrated quantum frequency processor on a silicon chip” describes an experimental realization of a integrated quantum frequency processor (QFP) on silicon photonics, with explicit classical demonstrations of using the QFP for single frequency encoded gate transformations like a tunable beam splitter and arbitrary rotations. Furthermore, the authors proceed to use part of their system to demonstrate the generation of a BPFC and perform coherent quantum walks. Their experimental results are excellent and are well supported by the existing theory or that which has been presented in the literature. We appreciate novel calibration methods that were presented. Overall we feel this manuscript is well suited for publication in Nature Communications after addressing our questions and comments.

1. The calibration procedure is very interesting, and I have a few questions there: (1) since you do this sequentially, for a

fixed EOM drive and desired WS phase, how long does the process take? After setting a WS channel, how many times do you need to go back to reset because of crosstalk? (2) You mention distinct dither tones can be applied to each WS ring, and that means you can perform Fourier analysis with one detected signal – how far detuned can you go before the modulation is too weak? (3) Practically, are you able to generate this dither from you source driving the rings, or do you need to multiplex the dither signals?
2. What is the total loss of the QFP (maybe include loss of the AMZI), and can this be stated clearly?
3. Based on the propagation losses (Qi of ~3e5 from BPFC at critical coupling) and loaded quality factors (Q of the filters in the WS ~5e4), 6dB insertion loss seems higher than expected. Can you comment on specifically where the loss is coming from? Do the phase shifters cause any loss?
4. Can you comment on what is required to increase the AMZI pump rejection and how that would affect the losses in the system? While maybe sufficient to limit parasitic SFWM in the waveguides, it seems like DWDMs and BPFs were required for the quantum walk experiments to further remove pump light.
5. Besides lowering loss, what are the next steps to make such a technology useful for quantum information processing? For instance, what is the estimated level of pump suppression one would need for impactful quantum frequency protocols with on-chip detectors? In turn, how would that impact loss? More discussion on these topics could be helpful for orienting the general quantum reader.

Reviewer #4

(Remarks to the Author)
This manuscript presents a fully integrated quantum frequency processor on a silicon chip, comprising of a photon pair source, a pump rejection filter, a four-channel line-by-line wave shaper, and a pair of high-speed phase modulators. Based on these elements, frequency-bin based quantum information processing is demonstrated, including tunable frequency beamsplitters, control of two-photon quantum walk, and on-chip quantum state tomography validation. While quantum information processing using frequency bins, the so call quantum frequency processor (QFP), has been theoretically studied and experimentally explored, it remains challenging to move all the elements on a single chip. This is due to the complexity of manipulating frequency bins that typically requires a phase modulator for spectral mixing and a line-by-line wave shaper for phase encoding, which means choosing the right material platform that can achieve both high speed modulation and large enough phase shift. In this sense, realizing a fully integrated QFP chip is impressive, despite the limited channel numbers and modulation speed might be a limiting factor for scalability. Overall, this is a great work with breadth demonstrations on the frequency bin information processing applications on a chip, the presentation is nice and clear. I have several comments for the authors.
1. Although the waveshaper channel design can leverage different operation states, given the 6dB insertion loss for each channel in PHASE state, will this structure introduce interference between the light being add-drop and directly passing through? This interference has already been observed shown in Fig. 2 in the Supplementary, distorting the transmission spectrum. How will it affect the frequency bin information processing? Intuitively, you are getting two sets of photons with different phases in PHASE state that is undesired.
2. Si chips are expected to have thermal crosstalk issues while using the thermal-optic tuning, how does this work deal with thermal crosstalk?
3. In the frequency beamsplitter demonstration, why the theoretical reflectivity and transmissivity are slightly different at $\alpha=\pi$?
4. For the reconstructed scattering matrix, can the authors add a brief description of how does it being constructed when first introduce it?
5. What is the FSR of the microresonators in wave shaper channels?
6. What is the accuracy of the phase control of wave shaper?
7. In the discussion section, can the authors comment on how to scale up the channel number using a monolithic chip, e.g. by reducing the frequency bin spacing, etc.? Since one major advantage of photonic chip integration is its scalability, but right now the modulation speed of the silicon modulator is already running at its limit, it would be nice to discuss how to go beyond two frequency bins with the current chip design.

Version 1:

Reviewer comments:

Reviewer #1

(Remarks to the Author)
I co-reviewed this manuscript with one of the reviewers who provided the listed reports. This is part of the Nature Communications initiative to facilitate training in peer review and to provide appropriate recognition for Early Career Researchers who co-review manuscripts.

Reviewer #2

(Remarks to the Author)
The authors have answered all my questions. I have no more questions and support its publication.

Reviewer #3

(Remarks to the Author)
The thorough and detailed response of the authors, together with the complete set of corresponding revisions to the manuscript, lends confidence that the manuscript is ready to proceed towards publication.

Reviewer #4

(Remarks to the Author)
The authors have addressed my comments with more details included in the revised manuscripts. I would recommend it to be published in Nature Communications.

Open Access This Peer Review File is licensed under a Creative Commons Attribution 4.0 International License, which permits use, sharing, adaptation, distribution and reproduction in any medium or format, as long as you give appropriate credit to the original author(s) and the source, provide a link to the Creative Commons license, and indicate if changes were made.
In cases where reviewers are anonymous, credit should be given to 'Anonymous Referee' and the source.
The images or other third party material in this Peer Review File are included in the article's Creative Commons license, unless indicated otherwise in a credit line to the material. If material is not included in the article's Creative Commons license and your intended use is not permitted by statutory regulation or exceeds the permitted use, you will need to obtain permission directly from the copyright holder.
To view a copy of this license, visit https://creativecommons.org/licenses/by/4.0/

We thank the four reviewers for their constructive and positive evaluation of the manuscript. All provided comments and questions have been much appreciated and we have done our best to address them in this response document. We have carefully considered all the remarks and revised both the main manuscript and the supplemental document accordingly.

Reviewer #1 (Remarks to the Author):

I co-reviewed this manuscript with one of the reviewers who provided the listed reports. This is part of the Nature Communications initiative to facilitate training in peer review and to provide appropriate recognition for Early Career Researchers who co-review manuscripts.

Reviewer #2 (Remarks to the Author):

In the manuscript titled ''Fully integrated quantum frequency processor on a silicon chip'' by Sara Congia, et al, the authors report a monolithic integration of a complete quantum frequency processor (QFP) on a silicon photonic platform. The authors successfully unify four critical functional blocks on a single chip and demonstrate high-fidelity frequency beamsplitting and on-chip quantum state tomography. The authors also provide a detailed and robust methodology for aligning ring resonances and mapping heater power to spectral phase, and the draft is easy to read. Overall, I recommend its publication in Nature Communications, but I still have a few questions.

1. The on-chip pump rejection filter provides around 30 dB attenuation. In some actual applications, 30 dB is not enough to isolate the single photons, for example in spontaneous four wave mixing. Did the authors use other filters (like off chip filters in this experiment) to obtain CAR of 55?
   In the current QFP chip, the integrated pump filter (PF) was designed as a simple asymmetric Mach–Zehnder interferometer (aMZI) to limit parasitic SFWM in the long circuit waveguides and to provide additional input and output ports to access the circuit. We are aware that single-photon isolation would have required a more sophisticated design and careful mitigation of the scattered pump light (see Comments #5&6 of Reviewer #3). Therefore, additional off-chip filtering is used to characterize the source in the single-photon regime, as reported in the setup depicted in Supplementary Fig. 1(c) and also shown in Fig.1 of this response letter.

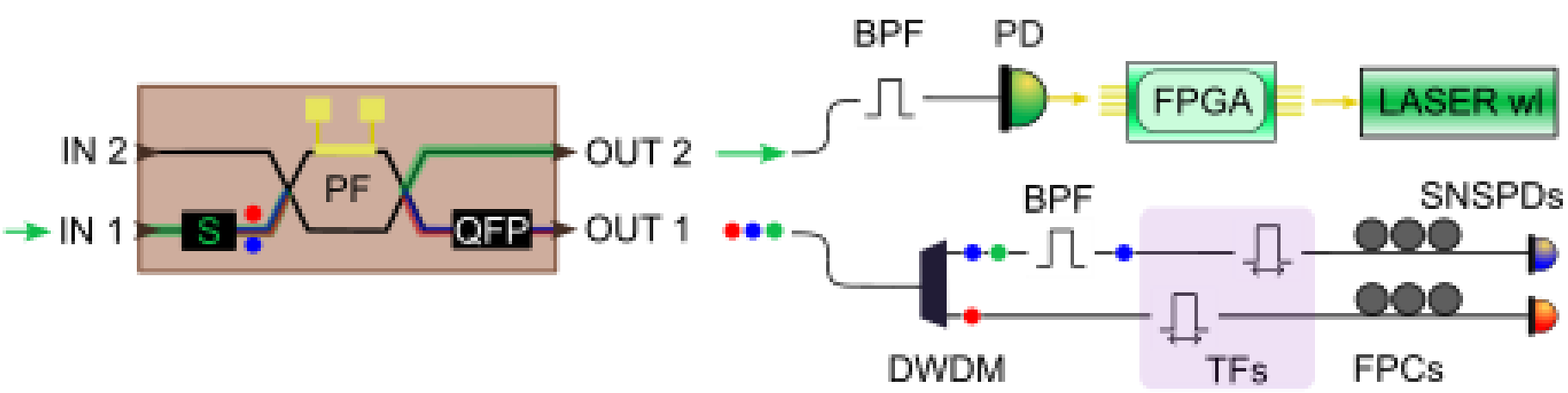

Figure.1. *Sketch of the filtering stage used to suppress the pump light from the signal and idler photons before frequency-resolved coincidence measurements.*

We will now describe how the source generation rate and CAR have been obtained, underlying the role of the PF and the frequency-resolving off-chip setup.

After the MRR source (S in the figure), the generated photon pairs and the attenuated pump (approximately 30 dB of attenuation) enter the lower arm of the aMZI-PF, and are directed to the rest of the circuit. During the characterization of the source in the photon-pair regime, the phase modulators are kept off and the waveshaper is detuned from the photon-pair frequencies. Photon pairs are then collected off-chip through OUT-1 and directed to a frequency-resolving coincidence setup. Here, the pump is attenuated by more than 100 dB, and idler and signal photons are separated and directed to distinct SNSPDs.
We recognise that in the previous version of the figure (and in the corresponding Methods section) the filtering elements were not sufficiently detailed. Therefore, the figure has been updated as shown in Fig.1 of this response letter.
We report here the list of filters, their extinction ratio (ER) and the 1dB bandwidth (BW):

- **DWDM**: Opneti optical WDM filter, channel 20 (centered at 1561.42 nm, at the signal bin wavelength). ER: 80 dB between non-adjacent ITU channels; BW: 80 GHz.
- **BPF**: tunable band-pass filter (Thorlabs TOF1550), centered at each idler frequency (around 1550.3 nm). ER: >60 dB; BW: 15 GHz.
- **TFs**: tunable filters based on fiber Bragg gratings. ER: >30 dB; BW: 12 GHz.

The internal generation rate and CAR as a function of the pump power, measured with the set of filters described above, are shown in Fig.2 of this response letter. The pump power implemented in the experiment is highlighted in yellow.

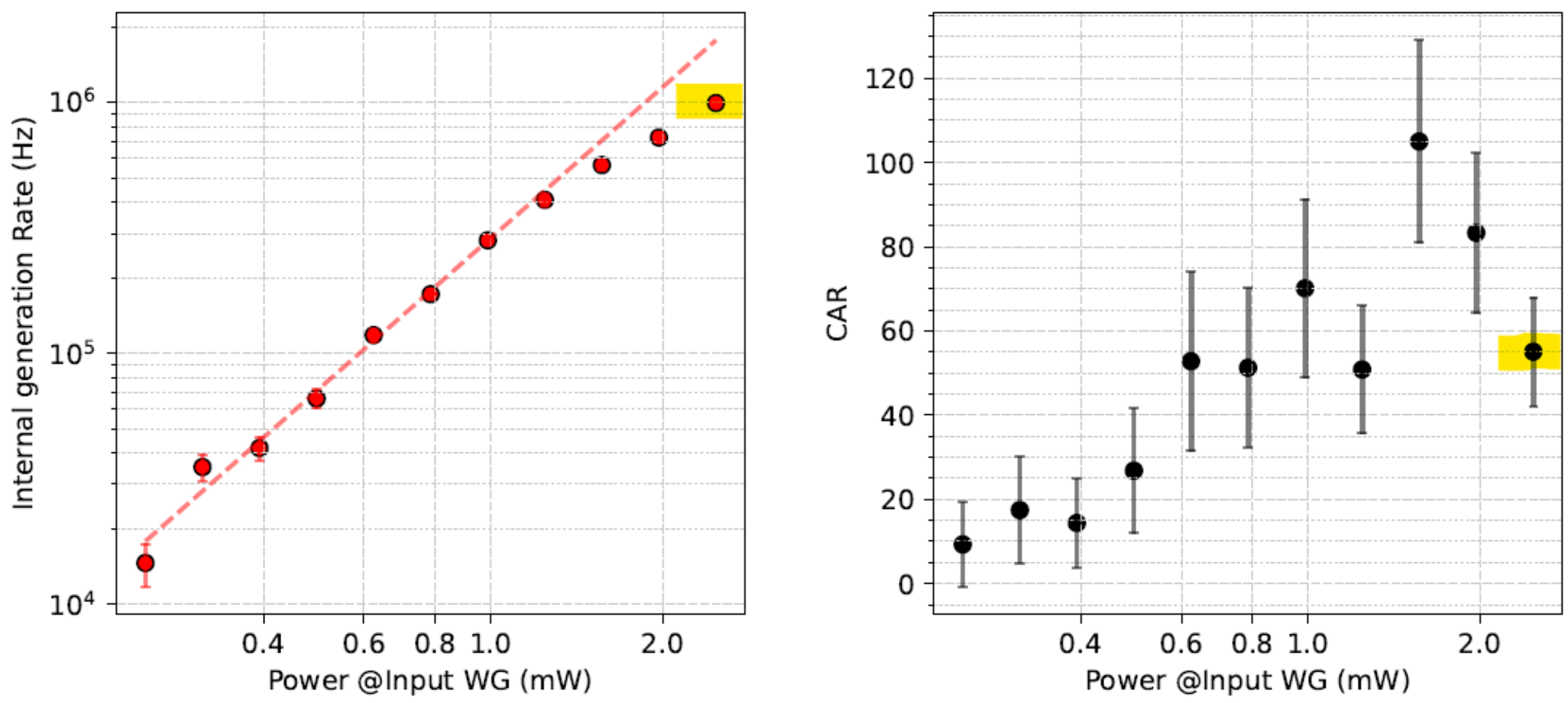

Figure.2. *Left(right), internal photon pair generation rate(CAR) as a function on the input pump power in the waveguide. The pump power used to reproduce the results in Fig.3 of the main manuscript is highlighted in yellow.*

Manuscript change:
In the main text, we have clearly stated that "*Additional off-chip filtering stages were implemented to perform coincidence measurements.*".
Figure 1 of the Supplemental document has been updated. In the Methods section of the revised version of the manuscript, section "Detailed description of the setup",

the extinction ratio and bandwidth of each off-chip filter has been specified to improve the reproducibility of the measurement in the photon pair regime.

2. Were there any observed effects of thermal crosstalk from the high-speed phase modulators during precise thermal tuning of cavity resonances? If not, can the authors add some theoretical analysis?
   High-speed PMs do indeed dissipate significant power through the resistive load of the electrodes, heating the entire chip. As a result, the ring resonances experience an overall shift of approximately 0.5 nm at the RF driving regime used in the experiment, with a small variation (<0.02 nm) between different ring pairs depending on their distance from the PM. For a given PM power, the shift of each ring can be recorded and compensated through the computer-controlled voltage driver.
   In our case, the dither-based calibration of the WS-channels was performed only after that the PMs were completely settled to their point of operation. The FFT peaks relevant for calibration lie in the 100–1000 Hz range; therefore, the 13-15 GHz driving frequency of the PMs does not influence the Fourier spectrum.
   The calibration must be repeated if the RF driving power is changed.

   Manuscript change:
   Methods section: "*Due to thermal crosstalk arising from the power dissipated by the phase modulators, calibration was performed while operating the modulators at the RF driving power of the experiment.*"

3. In the "STOP" configuration, one ring is aligned to the channel wavelength to block light. What is the measured extinction ratio for a single frequency bin in this state?
   The extinction ratio ranges from 12-15 dB, as reported in the main text.
   An example of transmission spectra of a WS channel in the STOP configuration is shown in Fig.3 of this response letter

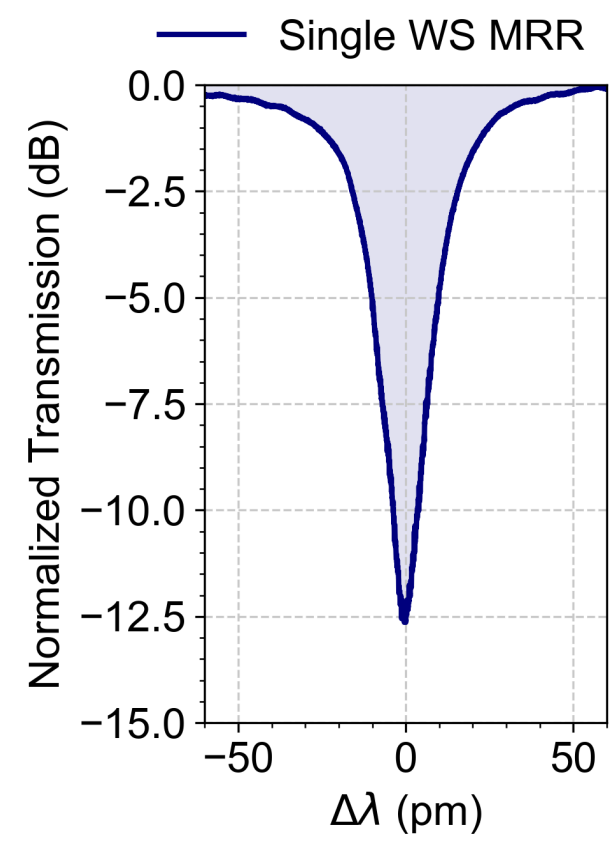

Figure.3. *Transmission spectra of the WS channel in the STOP configuration.*

   Manuscript change:
   We have included Fig.3 of this response letter in panel (a) of Fig.2 of the Supplemental document.

4. In the tomography experiment, spectral phases were applied to the idler modes while signal modes remained in the "PASS" configuration. Does this asymmetric projection scheme introduce any systematic bias in the reconstructed density matrix?

   In our current implementation, arbitrary single-qubit projectors can be applied to the idler photons, whereas the set of projectors available for the signal photons is limited to (in polarization notation) H (horizontal), V (vertical), D (diagonal), and A (anti-diagonal). Consequently, the tomographic set is not informationally complete. Nevertheless, the reconstructed density matrix of the two-qubit Bell state shown in Fig.3(e) exhibits a fidelity of 95.7(3)% with the target state, even with modest experimental statistics.

   This apparent contradiction can be explained by the fact that the target state is represented by a pure, sparse density matrix and is therefore characterized by fewer than the usual 15 independent real parameters representing arbitrary two-qubit states. For example, a pure two-qubit state described by a rank-1 matrix has only 6 independent real parameters.

   For such low-rank states, a resource-efficient approach for identifying the most informative measurement settings is threshold quantum state tomography [Binosi, Daniele, et al. "A tailor-made quantum state tomography approach." APL Quantum 1.3 (2024).]. In this method, one first determines the diagonal elements of the density matrix and, based on these outcomes together with a threshold parameter, selects the set of projectors that provides information about the most relevant density-matrix elements above the noise level. In principle, this set of projectors may be asymmetric; for example, it may include DR but not RD. To give a concrete example that directly applies to our case, we consider the set of projectors that bring direct information on the real and imaginary parts of the density matrix elements (Eq. 11 from Binosi, Daniele, et al. APL Quantum 1.3 (2024)):

$$\pi_2 = \begin{bmatrix} |HH\rangle & |HD\rangle + i|HR\rangle & |DH\rangle + i|RH\rangle & |DD\rangle + i|DR\rangle \\ 0 & |HV\rangle & |RR\rangle + i|RD\rangle & |DV\rangle + i|RV\rangle \\ 0 & 0 & |VH\rangle & |VD\rangle + i|VR\rangle \\ 0 & 0 & 0 & |VV\rangle \end{bmatrix}$$

   where the projectors are arranged such that the $Re(\rho_{ij}) = (Re(\pi_{2(i,j)}))\rho(Re(\pi_{2(i,j)})^{\dagger})$ and $Im(\rho_{ij}) = (Im(\pi_{2(i,j)}))\rho(Im(\pi_{2(i,j)})^{\dagger})$. For our target Bell state $(1/\sqrt{2})(|HH> + |VV>)$, the geometric mean of the elements $\rho_{22} = <HV|\rho|HV>$ and $\rho_{33} = <VH|\rho|VH>$, i.e. $\sqrt{\rho_{22}\rho_{33}}$, has been determined from the experimental data to lie below the user imposed threshold of $t = 0.01$ (see Fig.3(e)). According to Binosi, Daniele, et al. APL Quantum 1.3 (2024), all the elements of ρ which lie in rows and columns 2 and 3 are then set to zero. The remaining set of projectors to be measured are then DD and DR, which is asymmetric since it does not contain the projector RD.

After this selection, standard maximum-likelihood techniques employing a general unbiased parametrization of the density matrix, such as the Cholesky decomposition, are used to minimize the discrepancy between the expected and experimentally measured outcomes. In our case, the low-rank structure of the target density matrix enabled its faithful reconstruction from an asymmetric and tomographically incomplete set of projectors.

Manuscript change:
We added a brief summary of the above discussion in the revised version of the manuscript, and a new section in the supplementary materials *"DENSITY MATRIX RECONSTRUCTION WITH ASYMMETRIC PROJECTOR SETS"*

5. How is the overall loss in each component of the whole chip? Which component has the highest loss and how does it influence the ultimate performance?
The insertion loss of each component of the QFP has been probed at the wavelength of the pump at 1555 nm and they are listed in the table below

IN and OUT grating coupler	6.2 dB (3.1 each)
MRR source MZI-coupler	0.2 dB
2.8 cm-long circuit WG	3.4 dB (1.2dB/cm)
PF (maximum transmittivity)	0.2 dB
Phase modulators	3.0 dB (1.5 dB each)
**Total loss**	**13.0 dB**

Those losses are common to all frequency modes, including those passing through the on-chip WS. Frequency bins passing through the WS channels in the PHASE configuration experience an additional loss of ~6 dB, as already mentioned in the Methods section.

Manuscript change:
The insertion loss of the whole chip has been added in the Methods section. The losses of the individual components are also listed in the Methods section.

6. Based on current footprint and loss, what is the estimated maximum number of modes that could be realistically processed on a single wafer-scale chip?
We first discuss the three-element phase modulator (PM) / waveshaper (WS) / phase modulator architecture employed in this work. As discussed in the main text, this architecture can implement arbitrary operations on two frequency bins, although it is not universal over the four modes addressed by the integrated WS. Slightly higher fidelities and success probabilities for the single-qubit transformations could be achieved by increasing the number of WS units.

In the current design, adjacent WS units are separated by 1 mm-long bus waveguides, and the same separation is used between the two rings within each unit.

This spacing was chosen to mitigate thermal crosstalk between neighboring units. However, provided that thermal crosstalk is carefully managed, the separation could be significantly reduced, down to approximately $100\ \mu m$ per unit. Considering that the entire optical circuit currently occupies an area of 4.2 × 3.6 mm^2 (assuming the same modulator design is retained, i.e., PM length plus electrical routing to the RF pads, approximately 3.5 mm long), hundreds of WS units could in principle be integrated without increasing the die footprint.

Moreover, since each frequency bin passes through only a single WS unit, which constitutes the dominant source of loss, increasing the number of WS units would not substantially increase the overall insertion loss of the transformation, apart from the small additional contribution associated with propagation losses (approximately 0.01 dB over 100 μm of propagation).

More general transformations on ($N > 2$) modes can be achieved within a three-stage architecture by increasing the number of RF tones driving the PM (see e.g. Lu, Hsuan-Hao, et al. "Electro-optic frequency beam splitters and tritters for high-fidelity photonic quantum information processing." Physical review letters 120.3 (2018): 030502, and Lu, Hsuan-Hao, et al. "High-dimensional discrete Fourier transform gates with a quantum frequency processor." Optics Express 30.6 (2022): 10126-10134.). This approach does not introduce additional loss and does not alter the processor footprint.

At the highest level of complexity, however, arbitrary operations on N modes will likely require additional WS-PM stages. Unlike standard decompositions of arbitrary unitary matrices into beamsplitter networks, to the best of our knowledge there are currently no closed-form expressions for synthesizing arbitrary rotations over N modes using WS-PM blocks. Consequently, estimating the minimum number of blocks required to implement a given target transformation is nontrivial. Based on the decomposition of arbitrary unitary matrices into diagonal and circulant matrices (Lukens, Joseph M., and Pavel Lougovski. "Frequency-encoded photonic qubits for scalable quantum information processing." Optica 4.1 (2016): 8-16.), one can estimate an upper bound scaling as O(N) WS-PM blocks. Given the very small footprint achievable for a single WS unit, together with the size of each PM ($\sim 3000 \times 100\ \mu m^2$), tens of modes could in principle be manipulated within the current footprint, even in the worst-case scenario in which (N) WS-PM blocks are required. In such a case, however, the losses associated with each WS-PM block would need to be significantly reduced relative to the current design. While we acknowledge that achieving the same level of maturity as state-of-the-art universal photonic processors based on spatial encoding would be extremely challenging with the present technological platform (see, e.g., Taballione, Caterina, et al., “20-mode universal quantum photonic processor,” Quantum 7, 1071 (2023), which demonstrates a universal processor operating on 20 modes with only 1 dB transmission loss), the losses associated with each WS-PM block could nevertheless be reduced to approximately 1 dB through improved WS design strategies. Possible approaches include increasing the coupling strength between the ring resonators and the bus waveguide, as well as reducing both the ring-to-ring spacing and the separation between WS units.
However, we emphasize that one of the major strengths of frequency-bin

manipulation via electro-optic modulation lies in the possibility of driving the modulators with multiple RF harmonics. This approach has already been demonstrated to be highly effective for implementing high-dimensional Fourier gates, achieving dimensions up to 10 using only a three-element quantum frequency processor (Lu, Hsuan-Hao, et al., “High-dimensional discrete Fourier transform gates with a quantum frequency processor,” *Optics Express* 30, 10126–10134 (2022)). Therefore, increasing the number of driving harmonics while maintaining a limited physical circuit depth represents a highly promising strategy for reducing the overall loss of the processor.

Manuscript change:
We added parts of this discussion in the “*Discussion*” section of the revised manuscript.

Reviewer #3 (Remarks to the Author):

The work “Fully integrated quantum frequency processor on a silicon chip” describes an experimental realization of an integrated quantum frequency processor (QFP) on silicon photonics, with explicit classical demonstrations of using the QFP for single frequency encoded gate transformations like a tunable beam splitter and arbitrary rotations. Furthermore, the authors proceed to use part of their system to demonstrate the generation of a BPFC and perform coherent quantum walks. Their experimental results are excellent and are well supported by the existing theory or that which has been presented in the literature. We appreciate novel calibration methods that were presented. Overall we feel this manuscript is well suited for publication in Nature Communications after addressing our questions and comments.

1. The calibration procedure is very interesting, and I have a few questions there: (1) since you do this sequentially, for a fixed EOM drive and desired WS phase, how long does the process take? After setting a WS channel, how many times do you need to go back to reset because of crosstalk? (2) You mention distinct dither tones can be applied to each WS ring, and that means you can perform Fourier analysis with one detected signal – how far detuned can you go before the modulation is too weak? (3) Practically, are you able to generate this dither from you source driving the rings, or do you need to multiplex the dither signals?
   We outline here the procedure used to calibrate the waveshaper. Steps 1 and 2 are performed only once, while steps 3 and 4 are executed whenever the induced phase in the PHASE configuration needs to be changed.

   1. The resonances of the ring pairs within each WS unit are coarsely aligned to the target channel wavelength. The spectral alignment takes about **5 min**, and is obtained by sweeping the input laser and monitoring the transmission spectra with an oscilloscope.The heater powers P_M (MUX ring) and P_D (DEMUX ring) are saved. This initial setting already accounts for the main thermal crosstalk contributions from neighbouring WS units.
   2. A grid search is performed around P_M and P_D by varying the heater powers by ±5 mW (Fig.4(a) of this response letter). A first estimate of the peak position

of the $2(\Omega_D + \Omega_M)$ harmonic is obtained, and the heater power settings are updated accordingly. The wavelength span covered in this step is ~40 pm. The whole scan takes about **~20 min** for each unit, and the different units are sequentially scanned.

3. A second iteration is performed to compensate for the small additional thermal crosstalk induced after completing step 2. This time, a narrower and coarser grid search over a 10 pm wavelength range (Fig.4(b) of this response letter) is performed, and the new operating point (maximum of $2(\Omega_D + \Omega_M)$) is recorded. This scan takes about **~3 min** per unit. After this step, the resonances of each ring pair within the same unit are perfectly overlapped.
4. Whenever the WS phase needs to be changed, the power applied to the heater on the bus waveguides connecting the Drop and the Add port of the DEMUX and MUX ring is modified, and this induces a small thermal cross-talk to the microresonators within each unit. This is accomplished by performing the grid search described in **step 3 only once.**

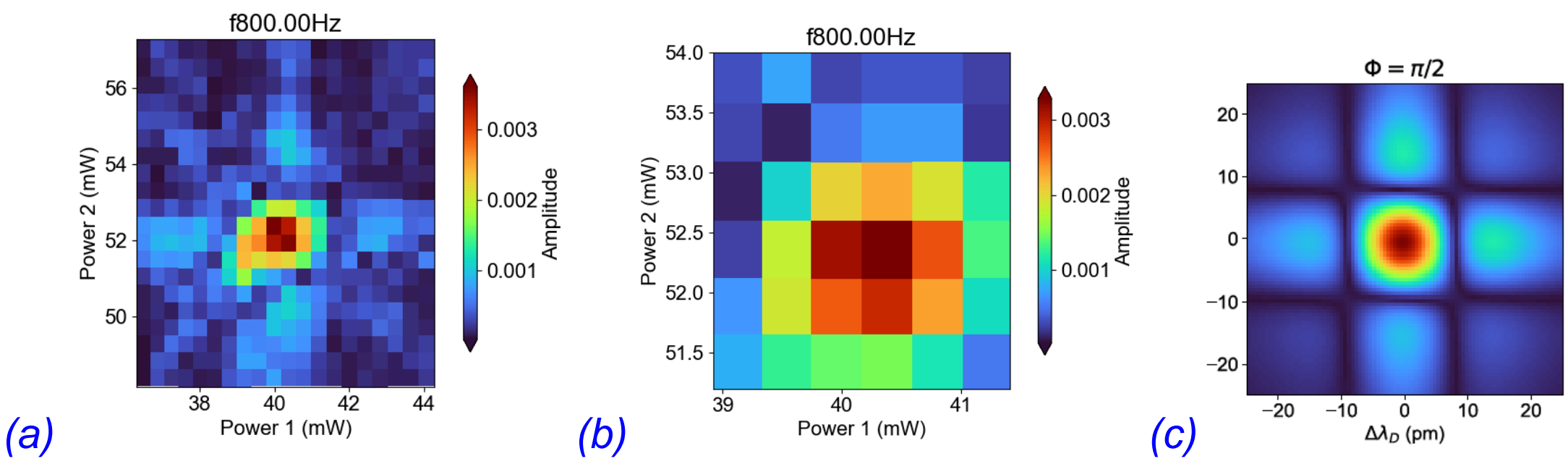

Figure.4. *Example of grid search applied to the heater powers during the waveshaper calibration. (a) Coarse grid search (step 2), showing the measured magnitude of the* $2(\Omega_D + \Omega_M)$ *harmonic in the transmitted intensity. (b) Narrow grid search (step 3) (c) Simulated magnitude of the* $2(\Omega_D + \Omega_M)$ *harmonic at the induced phase of* $\Phi = \pi/2$*, which matches the experimental pattern in panel (a).*

In summary, by taking into account the time required for the initial coarse alignment of all resonances to their target wavelengths (step 1, 5 min), the first grid search for each unit (step 2, 20 min × 4 = 80 min), and the final narrow search (step 3, 3 min × 4), the initial waveshaper calibration takes about **90 minutes**. After the initial calibration, every WS-unit phase change takes about 3 minutes (steps 3-4) to be completed.

The map of the intensity of the second harmonic amplitude shown in Fig.4(a) of this response letter shows that, for the dither amplitude chosen in the experiment, the Fourier component of interest is still 20-30 dB above the electronic noise (around -70dBm) in whole detuning range of 40 pm, which corresponds roughly to the FWHM of the resonances of the rings used in the WS unit. This range is sufficiently large to detect any possible residual misalignment of the resonances of the two rings, either

after the coarse alignment described in step 1 or induced by thermal cross-talk in the subsequent steps.

The two dither tones have been synthesized from a two-channel function generator, and added to the bias DC voltage of the heater driver controlling the microresonators by an external circuit.

Manuscript change:
In the Method section "*Calibration of the line-by-line waveshaper*", the calibration procedure has been described in more detail, emphasizing the two-distinct implemented grid searches. The time required to finish each step has also been reported.

2. What is the total loss of the QFP (maybe include loss of the AMZI), and can this be stated clearly?
   The insertion losses of the full circuit and of each component are listed in the answer to *Comment #5 of Reviewer #2*. These have been explicitly included in the revised version of the manuscript.

3. Based on the propagation losses (Qi of ~3e5 from BPFC at critical coupling) and loaded quality factors (Q of the filters in the WS ~5e4), 6dB insertion loss seems higher than expected. Can you comment on specifically where the loss is coming from? Do the phase shifters cause any loss?
   The 5~6 dB insertion loss of a WS unit is due to the limited *Drop* efficiency of WS' MRRs, which, as correctly pointed out by the reviewer, depend on both the WG propagation losses and the loaded Q-factor. We report in Fig. 5 of this response letter the simulation of the Through and Drop transmission spectra of a single MRR using the parameters (propagation losses and loaded Q) extracted from the experiment.

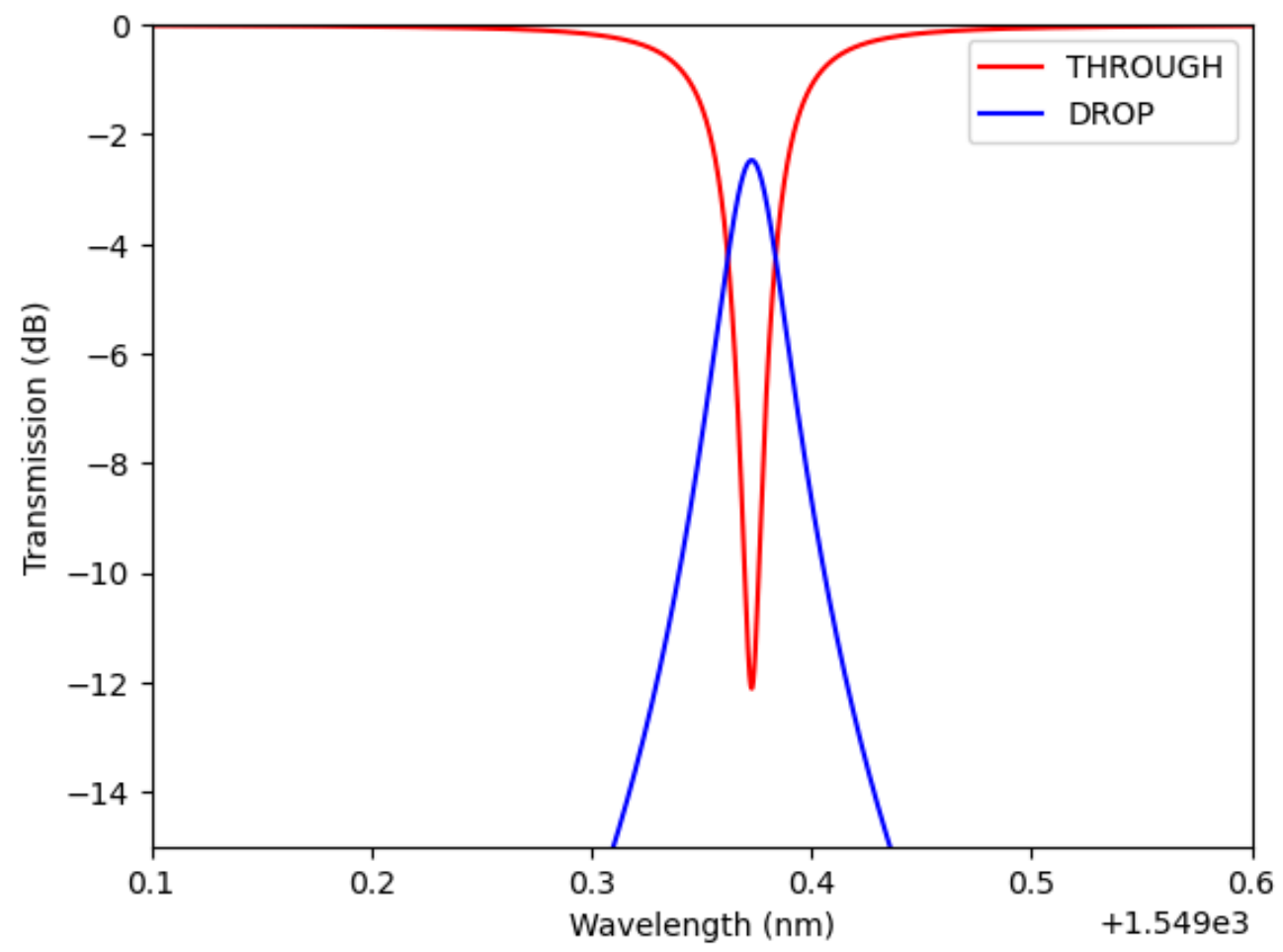

Figure.5. *Simulated spectral response of a single WS MRR.*

At resonance, the extinction at the Through port is -12dB while the intensity at the Drop port is ~2.5dB lower than that at the input. Within each WS unit, light at the channel wavelength (center of the resonance) is dropped by the DEMUX ring and later on reinjected into the common bus waveguide by the MUX ring, yielding a

combined loss of about 5 dB, which is very close to the losses measured in the experiment with the WS unit set in the PHASE configuration The WS unit features negligible loss outside the resonance bandwidth, as experimentally confirmed from the spectral response of the single MRR and of the WS unit (see Supplemental Document).
In conclusion, the 5~6 dB of insertion loss mentioned in the Method section are associated only to the frequency channel carried out by the unit and operated in the PHASE configuration.

Manuscript change:
To clarify the performances of the WS unit, the measured spectrum of a single WS MRR has been added in the Supplemental document (Fig.2(a), see also *Answer #3, Reviewer #2*). The origin of the losses of the WS unit in the PHASE configuration due to the finite drop efficiency is detailed in the Methods section.

4. Can you comment on what is required to increase the AMZI pump rejection and how that would affect the losses in the system? While maybe sufficient to limit parasitic SFWM in the waveguides, it seems like DWDMs and BPFs were required for the quantum walk experiments to further remove pump light.
More sophisticated filter designs will be required to fully suppress the pump light and eliminate the need for off-chip filtering. Using a single AMZI, the required suppression level, often quoted as exceeding 100 dB, cannot be achieved. However, cascaded AMZIs, commonly referred to as lattice filters, can provide both high extinction ratios and a high degree of control over the filter spectral response.

The total insertion loss of a lattice filter depends on the cumulative loss of its constituent components and therefore increases with the number of cascaded stages required to achieve the desired pump rejection. This also leads to a larger device footprint. In Ref. [Piekarek, Mateusz, et al., "High-extinction ratio integrated photonic filters for silicon quantum photonics," Optics Letters 42, 815–818 (2017)], the authors report several silicon-photonic lattice-filter designs with different numbers of cascaded stages to reach progressively higher pump suppression levels. An increase in insertion loss of approximately 6 dB is observed when moving from a simple two-stage lattice filter (ER = 30 dB) to an eight-stage design (ER > 70 dB).
Alternative filtering architectures can also be adopted and integrated on chip. In particular, CROW filters (coupled-resonator optical waveguides composed of cascaded microring resonators) are well known for their compact footprint and low insertion loss, albeit at the expense of a narrower operational bandwidth. In Ref. [Kumar, Rakesh Ranjan, Xinru Wu, and Hon Ki Tsang, "Compact high-extinction tunable CROW filters for integrated quantum photonic circuits," Optics Letters 45, 1289–1292 (2020)], CROW filters exhibiting insertion losses as low as 1.3 dB and extinction ratios exceeding 70 dB are reported, highlighting their potential for fully integrated quantum photonic processing.

To date, the work in Ref.[PsiQuantum team, "A manufacturable platform for photonic quantum computing," Nature 641, 876–883 (2025)] constitute the first demonstration of on-chip pump suppression to levels that exceed 100 dB, and is based the on silicon nitride platform and combined AMZI-add-drop resonator architectures.

Insertion losses as low as 1 dB at the signal and idler wavelengths have been reported in this work . These results demonstrate that the insertion losses associated with high-extinction filtering can be substantially reduced.

The AMZI was integrated into our circuit for two primary purposes. First, it acts as a pump filter, preventing spurious photon-pair generation via SFWM along the long bus waveguide of the circuit. Second, it provides additional on-chip routing capabilities that facilitate device testing and calibration. For example, light can be injected directly into one of the input ports of the PF, bypassing the microresonator source and enabling independent characterization of the frequency processor.

Manuscript change
In the revised manuscript, we have included additional details regarding the off-chip filtering stage (see the response to Reviewer #2, Comment #1) to clarify that off-chip filtering remains necessary for all characterizations and demonstrations performed in the photon-pair regime.

5. Besides lowering loss, what are the next steps to make such a technology useful for quantum information processing? For instance, what is the estimated level of pump suppression one would need for impactful quantum frequency protocols with on-chip detectors? In turn, how would that impact loss? More discussion on these topics could be helpful for orienting the general quantum reader.
   We thank the reviewer for the stimulating discussion. The integration of superconducting nanowire single-photon detectors (SNSPDs) in Si-based waveguides is technologically challenging, despite recent demonstrations even at the 200mm wafer scale (see Ref. [*Raj, Vidur, et al. "Waveguide integrated superconducting nanowire single-photon detectors for integrated photonics." Journal of Physics D: Applied Physics 58.24 (2025): 243001.*]). Beyond detector integration, a central challenge is the suppression of all the background noise photons that are not useful for quantum information processing, such as residual pump light, fluorescence and Raman scattering, which could otherwise trigger accidental detection events. As discussed in the previous answer, low-loss, high-extinction-ratio filters are crucial for pump rejection, while on-chip idler-signal demultiplexers are necessary to enable on-chip coincidence detection. Both components are compatible with standard photonic integrated circuit platforms and could be included in future processor designs.
   However, if extinction ratios exceeding 100 dB can be achieved while maintaining acceptable insertion loss at the photon-pair frequencies (<2 dB is a reasonable target, as discussed in the previous answer), this may still be insufficient for fully integrated on-chip quantum frequency-bin processing which includes detection. A well-known challenge in quantum photonic integrated circuits is scattered light, which bypasses the filtering stages and directly reaches the on-chip detectors. Waveguide loss, coupler loss, and any residual light leakage from the input fiber to the chip input may contribute to the background noise floor distributed across the chip volume [*Silverstone, Joshua W., et al. "Silicon quantum photonics." IEEE Journal of Selected Topics in Quantum Electronics 22.6 (2016): 390-402.*].
   To our knowledge, the lowest level of scatter rejection demonstrated to date is 115

dB with on-chip SNSPDs, reported in the Supplementary Information of Ref. [*PsiQuantum team. “A manufacturable platform for photonic quantum computing.” Nature 641, 876–883 (2025)*], achieved by surrounding the detectors with deep metal-filled trenches. Prior to this result, all experiments combining on-chip SFWM sources with on-chip filtering required the use of two separate chips to suppress the background noise from scattered light [*Kumar, Rakesh Ranjan, Xinru Wu, and Hon Ki Tsang. "Compact high-extinction tunable CROW filters for integrated quantum photonic circuits." Optics Letters 45.6 (2020): 1289-1292. , Kumar, Rakesh Ranjan, Xinru Wu, and Hon Ki Tsang. "Compact high-extinction tunable CROW filters for integrated quantum photonic circuits." Optics Letters 45.6 (2020): 1289-1292.*].

We therefore believe that implementing fully integrated quantum frequency-bin protocols with on-chip detection will require both careful filter design, comprising pump rejection and idler-signal demultiplexing, and dedicated scattering-attenuation architectures preceding the detectors. In the revised manuscript, we have expanded the Discussion section to include a discussion of these two functionalities as key directions toward fully scalable, chip-integrated quantum frequency processors .

Manuscript change:
The following sentence has been included at the end of the Discussion section in the revised version of the manuscript: “Promoting our architecture to a fully monolithic platform for quantum information processing, free of external optical components, will require the integration of high-extinction-ratio pump filters, signal–idler demultiplexers, and ultimately single-photon detectors together with dedicated optical isolation and scattering-attenuation structures. All of these building blocks have already been demonstrated in silicon-based photonic platforms [*PsiQuantum team. “A manufacturable platform for photonic quantum computing.” Nature 641, 876–883 (2025)*].“
The detailed discussion and the review of the strategies for pump and scattering suppression reported in this letter have been omitted to keep the manuscript compact and concise.

Reviewer #4 (Remarks to the Author):

This manuscript presents a fully integrated quantum frequency processor on a silicon chip, comprising of a photon pair source, a pump rejection filter, a four-channel line-by-line wave shaper, and a pair of high-speed phase modulators. Based on these elements, frequency-bin based quantum information processing is demonstrated, including tunable frequency beamsplitters, control of two-photon quantum walk, and on-chip quantum state tomography validation. While quantum information processing using frequency bins, the so call quantum frequency processor (QFP), has been theoretically studied and experimentally explored, it remains challenging to move all the elements on a single chip. This is due to the complexity of manipulating frequency bins that typically requires a phase modulator for spectral mixing and a line-by-line wave shaper for phase encoding, which means choosing the right material platform that can achieve both high speed modulation and large enough phase shift. In this sense, realizing a fully integrated QFP chip is impressive, despite the limited channel numbers and modulation speed might be a limiting factor for scalability. Overall, this is a

great work with breadth demonstrations on the frequency bin information processing applications on a chip, the presentation is nice and clear. I have several comments for the authors.

1. Although the waveshaper channel design can leverage different operation states, given the 6dB insertion loss for each channel in PHASE state, will this structure introduce interference between the light being add-drop and directly passing through? This interference has already been observed shown in Fig. 2 in the Supplementary, distorting the transmission spectrum. How will it affect the frequency bin information processing? Intuitively, you are getting two sets of photons with different phases in PHASE state that is undesired.

   The PHASE operation-related losses arise from the limited drop efficiency of the DEMUX and MUX ring resonator within the WS unit, and their origin are discussed in detail in Comment #3 of Reviewer #3.

   At resonance, the light transmitted to the through port of each ring is attenuated by approximately 12–15 dB (see Fig.5 of this response letter and the microring resonator spectra now added in the Supplementary document). Therefore, the light propagating directly through the bus waveguide of the WS unit, i.e., transmitted through the DEMUX ring and subsequently through the MUX ring, experiences >24 dB of total attenuation. In contrast, light that is dropped by the DEMUX ring and re-inserted into the common bus waveguide by the MUX ring, experiences approximately a loss of 5–6 dB (~2.5 dB of loss per drop event, see the simulated drop spectrum in Comment #3, Reviewer #3).

   Therefore, at resonance the light after the WS unit set into the PHASE comes exclusively from a single path. This situation changes out of resonance, where amplitude of the two interfering paths becomes comparable. The interference produced the distortions in the spectral response shown in the experimental WS spectra in Fig. 2 of the Supplementary document.

   However, as long as the bandwidth of the injected photons is small compared to that of the microrings in the WS unit, this does not constitute a concern for the correct WS operation. In our case, the Q-factor of the microrings is 50,000, while that of the photon-pair source resonator is Q~300,000, i.e. the bandwidth of the WS unit is 5 times higher than that of the input photons. Therefore, the phase shift experienced by the whole single photon wavepacket can be considered uniform over all its spectral components. Based on these arguments, we have modeled the WS operation on the frequency-mode $|j\rangle$ as $e^{i\Phi}|j\rangle$. The strong agreement (>98%) between experimental results and simulations (Fig.3(b) of the main manuscript) confirms that this assumption is well justified in our case.

   Manuscript change:

   We have included this discussion, i.e. on neglecting those channel photons passing through the corresponding WS-units and on the relative bandwidth of the source and WS rings, in the sections "WAVESHAPER CHARACTERIZATION AND SIMULATION" and "QUANTUM WALK SIMULATION" (supplementary document).

   "photons at the target channel wavelength are dropped by the two MRRs and experience the phase shift Φ in the waveguide section located between them. The alternative path, in which photons remain in the bus waveguide and pass through

both resonators, is more than two orders of magnitude less probable owing to the relatively high through-port extinction ratio at resonance (>12 dB per MRR).”
*“Note that all photons of a single frequency mode are assumed to experience a uniform phase-shift across their narrow frequency band. This is justified by the >5 times higher band*width of the WS MRR with respect to the idler and signal resonances.”

2. Si chips are expected to have thermal crosstalk issues while using the thermal-optic tuning, how does this work deal with thermal crosstalk?
   The calibration strategy for the MRR-based WS presented in the manuscript specifically addresses and corrects for the thermal crosstalk between the microring resonators. The detailed calibration procedure is described in #Comment 1 #Reviewer 3 and a brief summary is presented in the subsection “Calibration of the line-by-line waveshaper” of the Methods section.

   Manuscript change:
   We have underlined in the main text that the calibration procedure was indeed used to face the thermal crosstalk issue: “The procedure (detailed in the Methods section) allows optimizing the WS settings despite the presence of thermal crosstalk between the components.”

3. In the frequency beamsplitter demonstration, why the theoretical reflectivity and transmissivity are slightly different at $\alpha=\pi$?
   We assume that the reviewer is referring to the plot in Fig. 2c (main text). The solid curves fit the experimental data points. The data were fitted using Eq.(3) of the Supplemental document using the modulation depth δ as a free parameter. The fit considers both y-errors (<0.1% arising from the uncertainty on the measured BS splitting ratio) and x-errors (from the conversion between the heater power to the induced phase α). The shaded region in the plot represents the 95% confidence band of the fit.
   The resulting fit yields $\delta = 0.843 \pm 0.006$, consistent with the modulation index of $0.82 \pm 0.02\pi$ extracted by fitting the intensity of the first order sidebands as a function of the applied RF power. Since the modulation index slightly differs from that producing perfectly balanced reflection and transmission, the two are not equal at $\alpha = \pi$.

   Manuscript change:
   We further emphasized that the shaded regions refer to a *fit* of the data in which the modulation index has been used as a free parameter in the caption of the figure.

4. For the reconstructed scattering matrix, can the authors add a brief description of how does it being constructed when first introduce it?
   The procedure for the characterization of the scattering matrix closely follows the lines described in [Rahimi-Keshari, S. et al. Direct characterization of linear-optical networks. Opt. Express 21, 13450–13458 (2013)], and implemented on the frequency bin platform in [Lu, Hsuan-Hao, et al. "Electro-optic frequency beam splitters and tritters for high-fidelity photonic quantum information processing." Physical review letters 120.3 (2018): 030502].

Manuscript change:
We added a new section in the Supplementary Materials "Characterization of the beamsplitter scattering matrix" where this method and its experimental implementation are described in detail.

5. What is the FSR of the microresonators in wave shaper channels?
We thank the reviewer for having spotted this missing information in the manuscript. The WS's MRRs are all nominally identical, featuring a FSR of 250GHz.

Manuscript change:
We have added this information in the Method section "*Detailed description of the processor layout*".

6. What is the accuracy of the phase control of wave shaper?
The phase control accuracy of the waveshaper is inferred from the uncertainty of the parameters $P_{2\pi}$ and ϕ_0 in the cosine fit applied to the real part of the harmonic $\Omega_M - \Omega_D$ (see panel description of Fig.3(g)). We found that the dominant source of inaccuracy lies in $P_{2\pi}$ ($\delta P_{2\pi} \sim 1\ mW$), corresponding to a phase uncertainty of 6°.

Manuscript change:
We have now added the value of $P_{2\pi} \pm \delta P_{2\pi}$ and the phase accuracy in the caption of Fig.1(g) (main text).

7. In the discussion section, can the authors comment on how to scale up the channel number using a monolithic chip, e.g. by reducing the frequency bin spacing, etc.? Since one major advantage of photonic chip integration is its scalability, but right now the modulation speed of the silicon modulator is already running at its limit, it would be nice to discuss how to go beyond two frequency bins with the current chip design.
We thank the reviewer for this comment. We had not explicitly discussed the possibility of increasing the channel count by reducing the frequency-bin spacing, which is indeed a viable approach. However, the minimum achievable channel spacing is ultimately constrained by several physical and technical factors, as discussed below.

Bandwidth of the microring resonators within the WS channels. For frequency-bin spacings comparable to the FWHM of the microring resonators, the WS unit cannot efficiently separate and route the target frequency bins without introducing significant spectral crosstalk. As shown by both the experimental and simulated transmission spectra of the microresonators (see Comment #3, Reviewer #2), the through-port transmissivity of a single WS MRR returns to its maximum value only approximately 70 pm away from resonance. Consequently, the channel spacing can be reduced by at most a factor of two compared to that used in the present experiment (120 pm, corresponding to 15 GHz). Achieving smaller frequency-bin spacings would therefore require microresonators with higher Q-factors, obtained by reducing the round-trip optical losses and optimizing the coupling coefficient to the bus waveguide accordingly.

Notably, integrated line-by-line WS systems with channel spacings as small as 3 GHz have recently been demonstrated in silicon photonics [Cohen, Lucas M., et al., *Silicon photonic microresonator-based high-resolution line-by-line pulse shaping*, Nature Communications 15, 7878 (2024); Wu, Kaiyi, et al., *On-chip pulse shaping of entangled photons*, Physical Review Research 7, 033015 (2025)]. These implementations report channel insertion losses of approximately 6 dB, comparable to those achieved in our work. Further reductions in both insertion loss and channel spacing are expected from ultralow-loss integrated platforms such as silicon nitride.

Bandwidth of the photon pairs and FSR of the photon-pair source. The free spectral range of the microresonator-based photon-pair source must match the frequency-bin spacing of the WS unit. Consequently, reducing the channel spacing requires a proportional increase in the source Q-factor to maintain a constant finesse and, therefore, a high photon-pair generation brightness. In addition, to ensure that the spectral phase applied by each WS unit remains approximately uniform across the entire single-photon spectrum, the FWHM of the source microresonator resonance must remain significantly smaller than the bandwidth of the WS unit. Simultaneously satisfying these two requirements places stringent demands on the Q-factor of the source resonator and therefore necessitates a further reduction in cavity losses. An alternative strategy to overcome the trade-off between high brightness and low FSR is the use of microresonator arrays, consisting of multiple nominally identical high-finesse resonators [Clementi, Marco, et al., *Programmable frequency-bin quantum states in a nano-engineered silicon device*, Nature Communications 14, 176 (2023); Pang, Yiming, et al., *Versatile chip-scale platform for high-rate entanglement generation using an AlGaAs microresonator array*, PRX Quantum 6, 010338 (2025)]. In this architecture, the photon-pair generation brightness is effectively decoupled from the FSR, which can be continuously engineered by introducing controlled offsets between the resonance combs of the individual rings, potentially reducing the effective spacing down to the GHz regime.
Using two nominally identical MRRs, an effective FSR down to 1 GHz has already been demonstrated in an ultra-low-loss silicon nitride photonic chip (Ref. *[Vinet, S., et al. Time-resolved certification of frequency-bin entanglement over multi-mode channels. npj Quantum Inf 12, 38 (2026)]*), confirming that the combined strategy of reducing propagation losses and using engineered sources is a viable approach toward scalable frequency-domain photonic architectures.

Manuscript change:
We have added this possibility of scaling-up the number of channels by reducing the frequency spacing in the revised version of the manuscript.
“Further scaling of the processor's information capacity could be achieved by reducing the frequency bin spacing, which would require a reduction in the round-trip optical losses of the MRRs and adapting the source design accordingly, with ultralow-loss platforms such as silicon nitride representing a promising pathway toward this goal.”
We have also added a discussion on the number of modes that can be realistically addressed within the footprint of the current device. In particular, we highlight the main scalability limitations, identify potential approaches for reducing the insertion loss of individual components, and outline strategies for increasing the information

capacity of the processor without increasing its physical depth through the multiplexing of multiple RF driving harmonics.
